# Single Cell RNA-Seq and Machine Learning Reveal Novel Subpopulations in Low-Grade Inflammatory Monocytes With Unique Regulatory Circuits

**DOI:** 10.3389/fimmu.2021.627036

**Published:** 2021-02-23

**Authors:** Jiyoung Lee, Shuo Geng, Song Li, Liwu Li

**Affiliations:** ^1^Ph.D. Program in Genetics, Bioinformatics and Computational Biology, Virginia Polytechnic Institute and State University, Blacksburg, VA, United States; ^2^School of Plant and Environmental Sciences, Virginia Polytechnic Institute and State University, Blacksburg, VA, United States; ^3^Department of Biological Sciences, Virginia Polytechnic Institute and State University, Blacksburg, VA, United States

**Keywords:** monocyte, machine learning, inflammation, regulatory motifs, single cell analysis

## Abstract

Subclinical doses of LPS (SD-LPS) are known to cause low-grade inflammatory activation of monocytes, which could lead to inflammatory diseases including atherosclerosis and metabolic syndrome. Sodium 4-phenylbutyrate is a potential therapeutic compound which can reduce the inflammation caused by SD-LPS. To understand the gene regulatory networks of these processes, we have generated scRNA-seq data from mouse monocytes treated with these compounds and identified 11 novel cell clusters. We have developed a machine learning method to integrate scRNA-seq, ATAC-seq, and binding motifs to characterize gene regulatory networks underlying these cell clusters. Using guided regularized random forest and feature selection, our method achieved high performance and outperformed a traditional enrichment-based method in selecting candidate regulatory genes. Our method is particularly efficient in selecting a few candidate genes to explain observed expression pattern. In particular, among 531 candidate TFs, our method achieves an auROC of 0.961 with only 10 motifs. Finally, we found two novel subpopulations of monocyte cells in response to SD-LPS and we confirmed our analysis using independent flow cytometry experiments. Our results suggest that our new machine learning method can select candidate regulatory genes as potential targets for developing new therapeutics against low grade inflammation.

## Introduction

The innate immune system acts as the first and immediate line of defense targeting broad pathogens through leukocytes such as neutrophils and monocytes. Lipopolysaccharide (LPS), also known as bacterial endotoxin, is a key danger-signal causing various inflammatory responses from the host (1, 2). A higher dose LPS often accompanying with acute bacterial infection can trigger a robust yet transient inflammatory response, coordinating pathogen removal, and tissue homeostasis (3). In contrast, subclinical doses of LPS commonly associated with chronic inflammation and mucosal leakage can cause low-grade and chronic inflammation (1–4). Moreover, chronic inflammation contributes to diseases such as cancers, atherosclerosis, and other metabolic syndromes (1, 2, 5, 6). However, the mechanism of innate immune responses under chronic and low-grade inflammation is poorly understood.

There have also been attempts to intervene with the establishment of low-grade inflammation through the application of compounds such as 4-PBA that is a fatty acid molecule and known as a chemical chaperon in ER stress and a histone deacetylase inhibitor (7–9). The important clinical application of 4-PBA has been reported as a treatment for children's urea cycle disorders (8). Also, other therapeutic potential and effects of 4-PBA have been investigated from murine model for neurodegenerative diseases such as Alzheimer's disease (8). In particular, previous studies explored how 4-PBA affects chronic inflammation induced by subclinical exotoxin in macrophage and neutrophils, and reported that 4-PBA restores anti-inflammatory responses in the innate immune system (7, 9). These findings provide a novel insight about 4-PBA as a promising therapeutic compound for chronic inflammatory diseases. The goal of this study is to understand the underlying mechanisms of innate immune responses toward low-grade inflammation and to use this knowledge to contribute to the understanding and treatment of chronic inflammatory disease.

Single cell RNA-seq (scRNA-seq) has been widely used to profile gene expression in individual cells, and overcomes limitations of bulk RNA-seq (10–13). The overall objective of this study is to identify the different states and key regulators within the inflammatory responses that are super-low dose-specific in monocytes. Although a number of studies have analyzed the response of monocyte to high dose LPS using single cell RNA-seq technology (14, 15), there is no prior study using scRNA-seq to characterize monocyte populations under super-low dose LPS. Whether monocytes, a relatively homogenous cell population, can be further categorized into sub-populations under such low dose of external stimuli is still an open question. In order to achieve this objective, we generated scRNA-seq data from monocytes with super-low dose LPS (SLD-LPS, 100 pg/ml), 4-PBA and a mixture of super-low dose LPS and 4-PBA treatments and employed the following strategies. First, we performed single cell clustering analysis using dimensional reduction and visualized cells into subpopulations (13, 14). Second, we examined the gene expression profile of known and potential marker genes associated with specific immune responses in monocytes. Third, we applied single cell trajectory inference to predict transitional cell populations (13, 14, 16, 17). While it is only possible to trace transcriptional changes with time series data in bulk RNA-seq, trajectory analyses in scRNA-seq reconstruct pseudo-time axis and infer predicted transitions of cell states (11, 13, 15–17). Finally, we identified potential gene regulatory relationships between transcription factors and target genes from scRNA-seq and ATAC-seq using a machine learning method (13, 18).

From the analysis of our scRNA-seq data, we have discovered that super-low dose LPS induced two subpopulations of low-grade inflammatory monocytes which were not found with bulk RNA-seq data. We have identified unique and novel marker genes in these two states. We characterized functional annotations of these genes that show distinct changes along pseudo-time that reflected possible biological transition of cellular responses. Our analyses also reveal that 4-PBA challenge largely masked the effects of LPS. Using regulatory sequence analysis and machine learning, we identified 10 motifs that are potential key regulators of the dynamic immune responses to super low dose LPS and 4-PBA. Among these motifs, we have found evidence that STAT1/2 and IRFs are candidate regulatory genes involved in the activation process. These discoveries of novel sub-populations of monocytes, potential key regulators, and novel markers were validated in protein levels by using flow cytometry.

## Materials and Methods

### Sample Collection

Crude bone marrow cells were isolated from of 6–8 weeks old male C57BL/6 mice, and cultured in RPMI 1,640 medium supplemented with 10% fetal bovine serum, 2 mM L-glutamine, 1% penicillin/streptomycin and with monocyte colony stimulating factor (M-CSF, 10 ng ml^−1^) as we previously described (5). Cultured monocytes were treated as described and briefly listed as follows. (1) PBS: phosphate-buffered saline as a control; (2) SLD-LPS: super-low dose lipopolysaccharide (100 pg/ml); (3) 4-PBA (1 mM): 4-phenylbutyrate known as a potential therapeutic agent to reduce pro-inflammatory responses; and (4) 4-PBA and LPS: a mixed treatment with both SLD-LPS (100 pg/ml) and 4-PBA (1 mM). We used *E. Coli* 0111:B4LPS strain which has been shown to be solely dependent on TLR4 receptor (19, 20). Fresh LPS and 4-PBA were added to the cell cultures every 2 days, and the cells were harvested on day 5 to simulate *in vitro* chronic low-grade inflammation.

### Single-Cell Sequencing

The prepared cell samples were processed by 10X Genomics Chromium Single Cell 3' Reagent Kits (version 3 Chemistry) for scRNA-seq, and sequenced by Illumina platform. In brief, 5 × 10^5^ cells of each sample were sorted and then centrifuged at 300 g × 5 min, 4°C. The supernatant was aspirated, and cells were re-suspended in 100 μl of ice cold 1× PBS containing 0.04% BSA. The cells were then counted with trypan blue to make sure high viability above 98%. Cell concentration was adjusted to exactly 700 cells/μl by ice cold 1× PBS containing 0.04% BSA, and cells were kept on ice. Libraries were prepared using the 10X Genomics Chromium Single Cell 3' v3 Library and Gel Bead Kit. Single-cell suspensions were loaded onto the Chromium Controller to generate 1,000 single-cell gel beads in emulsion per sample. The cDNA of each sample after amplification of 12 cycles was quantified by Qubit and quality-checked by Bioanalyzer to verify the size distribution of cDNA samples and determine indexed PCR amplification of 15 cycles to yield a sufficient and unbiased library for sequencing. After library quality control by Tapestation, indexed library samples were quantified by KAPA library quantification kit (Universal) and pooled with 15 ul of 5 nM for each sample. Pooled library sample was sent to Novogen for sequencing. Pair-end sequencing was performed on Illumina^®^ HiSeq platform, with the read length of paired-end 150 bp at each end plus 8-bp i7 index. Cells collected from three individual mice were treated and then pooled together for library preparation and sequencing, in order to achieve robustness and to reduce batch variation.

### Raw Single Cell RNA-Seq Data

Raw sequencing data were analyzed using the Cell Ranger (version 3.0.2) with mouse reference genome and annotation (Cell Ranger reference version 3.3.0, mm10, Ensembl 93) from the 10X Genomics website (https://support.10xgenomics.com/single-cell-gene-expression/software). The Cell Ranger pipeline included mapping sequenced reads, and quantifying gene expression. Approximately, 2,800 cells were obtained from four samples.

### Clustering Analysis

Single cell data from four treatments were analyzed by Seurat (version 3.1.2) in R (13). Quality control, data normalization, and scaling were performed using default pipeline of Seurat. As a quality filtering step, cells with more than 20% of reads from mitochondrial genes, and cells that have more than 6,500 or fewer than 200 unique genes were removed. The individually samples were processed, and ~2,200 cells from four treatments were retained and merged. Data were normalized and scaled before dimensionality reduction which is performed by principal component analysis (PCA), and UMAP were used for clustering the cells using a graph-based clustering approach (13). We used UMAP for visualization, which is known to have better performance than other methods and reflect distances between cells in different clustering groups and within the same clustering group. With the clustering result, marker genes that are differentially expressed and that are expressed in at least 10% of cells in a target cluster were obtained for each cluster by using the non-parametric Wilcoxon rank sum test in R.

### Trajectory Analysis of Single Cell RNA-Seq

A trajectory analysis was performed through dyno workflow with dyno (version 0.1.1) and tidyverse (version 1.3.0) libraries in R (16). Dyno library provides a framework to facilitate decisions to select the best methods with available computing capacity and prior resources of users (16). Gene expression matrices, dimensionality reduction coordinates, clustering, and cell information were extracted from the clustering results then added during the inference process. A starting cell that can be considered as a root cell was decided among randomly selected cells, that belongs to a cluster with cells mainly from PBS treatment. Dynbenchmark was used to determine a set of methods with available resources, given prior information and data size. A minimum spanning tree (MST) was selected as a trajectory inference. In order to optimize the trajectory on the clustering results, MST script in dynverse github (https://github.com/dynverse/ti_mst/blob/master/run.R) was optimized by using the UMAP coordinates as input of mclust in R (17) to decide the best model for clustering and the number of components based on the highest BIC value. Trajectory was visualized on the UMAP coordinates. Predictive genes were differentially expressed genes and changed expression at or along a branch. The predictive were defined by a R package dynfeature, and visualized as a heatmap along the trajectory using the R package dynverse (16).

### ATAC-Seq Analysis

We obtained raw ATAC-seq reads of monocytes from GSE100738 (SRR5799491, SRR5799492, SRR5799494, SRR5799493, SRR5799541, SRR5799542) (18). Based on reports from FastQC (21), data sets are of high quality and trimming and filtering are not necessary. Reads were mapped to the mouse reference (mm10, Ensembl 89 GRCm38) using botwie2 (bowtie2 -k 4 -q -X 2,000 –local –mm –fr –no-unal) (22). Samtools (samtools view -F 1,804 -f 2 -q 30 -h -b) (23) and picard (picard MarkDuplicates with default options) (24) were used to select properly mapped reads with high mapping quality. Filtered reads were merged and peaks were called using HMMRATAC (java -jar $HMMRATAC with default options) (25). Peak annotation was performed with the open chromatin regions of peaks using annotatePeaks.pl in HOMER (26). The open chromatin regions that were annotated as the promoter-TSS type were used in our analysis. Ensemble gene accessions were converted into gene names to match names in scRNA-seq.

### Selection of Positive and Negative Training Sets

Genes for positive sets and negative sets for each cluster were defined according to the average fold change and statistical significance from scRNA-seq and annotation as target genes on open chromatin regions from ATAC-seq analysis. Genes for positive sets were first selected among up-regulated differentially expressed genes (DEG) for individual clusters, respectively where the minimum percentages of expressed cells of the genes were 10%, adjusted *p*-values of the genes were lower than 0.05, and the average fold changes of the genes in cells were equal to or >1.5-fold. The DEG that were annotated from the peak annotation were then retained in positive sets for individual clusters.

Genes for negative sets were first selected among non-DEG for individual clusters, respectively, where the minimum percentages of expressed cells of genes were still 10%, but adjusted *p*-values of the genes were higher than 0.1, and the abstract average fold changes of genes were smaller than 1.5-fold. Then the non-DEG were separated into two types of negative sets to compare performances of methods depending on whether the non-DEG were annotated on open chromatin regions or not by peak annotation. The first type of negative sets (negative set 1) included genes that were the non-DEGs and not annotated from the peak annotation (non-OCR). The second type of negative sets (negative set 2) included genes that were the non-DEGs and annotated from the peak annotation (OCR). Two types of negative sets were generated for each cluster for next step.

### Motif Search and Definition of Positive and Negative Gene Sets

Genomic sequences of the positive sets and negative set 2 were directly extracted from the coordinates of detected open chromatin regions. Genomic sequences of the negative set 1 were extracted from 1,000 bp upstream and to 220 bp downstream of transcriptional start sites (TSS) of genes in the negative set 1. When there were multiple TSS for a gene, we chose a longest protein coding transcript. Transcription factor binding site models of 531 mouse motifs (HOCOMOCOv11_full_MOUSE_mono_meme_format.meme) were obtained from HOCOMOCO v11 webpage (https://hocomoco11.autosome.ru/downloads_v11) (27). FIMO (meme-5.0.5 version) (28) with default parameters was used to scan the extracted regulatory sequences of gene sets for individual clusters with each of the HOCOMOCO mouse motifs. FIMO hits with *q* > 0.05 were counted by gene and motif and converted as a motif profile. Final positive gene sets, negative gene set 1, and negative gene set 2 were determined among genes with motifs on their regulatory sequences.

### Motif Enrichment Analysis and Feature Selection

Motifs from positive gene sets and two negative gene sets were used as features for individual clusters, respectively. Input data sets of the enrichment analysis were FIMO hit counts of individual motifs for genes in positive gene set, negative gene set 1, and negative gene set 2. Chi-squared test and random forest were applied to the motif count data to perform motif enrichment analysis and feature selection, respectively. For enrichment test, Chi-squared test (CT) with Yates' continuity correction was performed. Motifs at least 5 hits were applied for the test as following its requirement. *P*-values were calculated for individual motifs, and top 10 motifs by CT were selected based on the -log_10_ *p*-value for each cluster, respectively.

As another method to select features, a machine learning method called guided regularized random forest (GRRF) was used (29). To perform the guided regularized random forest, K-fold cross-validation on input data was conducted with *K* = 5. The input records were split into five subsets. Genes from negative gene sets and positive gene sets were sampled by the stratified random sampling method using create Folds function in R package Caret (30). One subset was assigned as a testing data set (20%), then among four subsets one subset was assigned as a validation data set (20%), and rest of three subsets were merged and assigned as a training data set (60%). Four combinations of training-validation data sets were created for one testing data set, and all five subsets were assigned as a testing data set once. To build a GRRF model, an ordinary random forest model was first trained on training data sets to obtain a list of max-normalized importance scores. GRRF models with different lists of weighted average coefficients were generated from different regularization gamma values on training-validation data sets to optimize this hyper-parameter. Accuracies across GRRF models with the different coefficients were compared, and a GRRF model with the highest accuracy on validation data was used to predict testing data sets. Performances of trained random forest models on validation data set were also evaluated by ACC (accuracy), MCC (Matthews correlation coefficient), F1 score (the harmonic mean of precision and recall). The best model of each iteration of training-validation data set was determined based on the maximum value of ACC × MCC × F1. Performances of prediction on testing data sets by the best models from four iterations were evaluated by auROC (the area under the receiver operating characteristics) and auPRC (the area under the precision recall curve). The best model of each cluster was decided based on auROC value. Selected features and their importance score (mean decrease in Gini Index) were obtained from the best model on training-validation data sets, and top 10 motifs by GRRF were selected based on the importance score for each cluster.

### Gene Set Enrichment Analysis or Functional Annotation

Functional enrichment analysis of groups of genes were performed by ShinyGO (version 0.61, Ensembl Biomart 96), an online gene-set enrichment tool searching annotation from various pathway databases such as gene ontology, KEGG pathway (31). Predictive genes from trajectory analysis were annotated by enriched functional categories from KEGG pathways with *p*-value cutoff (FDR) < 0.05 from mouse metabolic pathways (314 genesets, R.82.0).

### FACS Cytometry Experiment

Cells were stained with specific antibodies against CCR2 (MCP1), CCR5, CX3CR1, and C5AR1 (CD88) according to protocols we described previously (5). Labeled cells were analyzed by FACS Canto II flow cytometer and data analyzed with FloJo Software as we described previously (5). Data from three biological replicates were plotted, and representative of two independent repeats.

## Results

### Experimental Design and scRNA-Seq Quality Control

**Figure 1:**
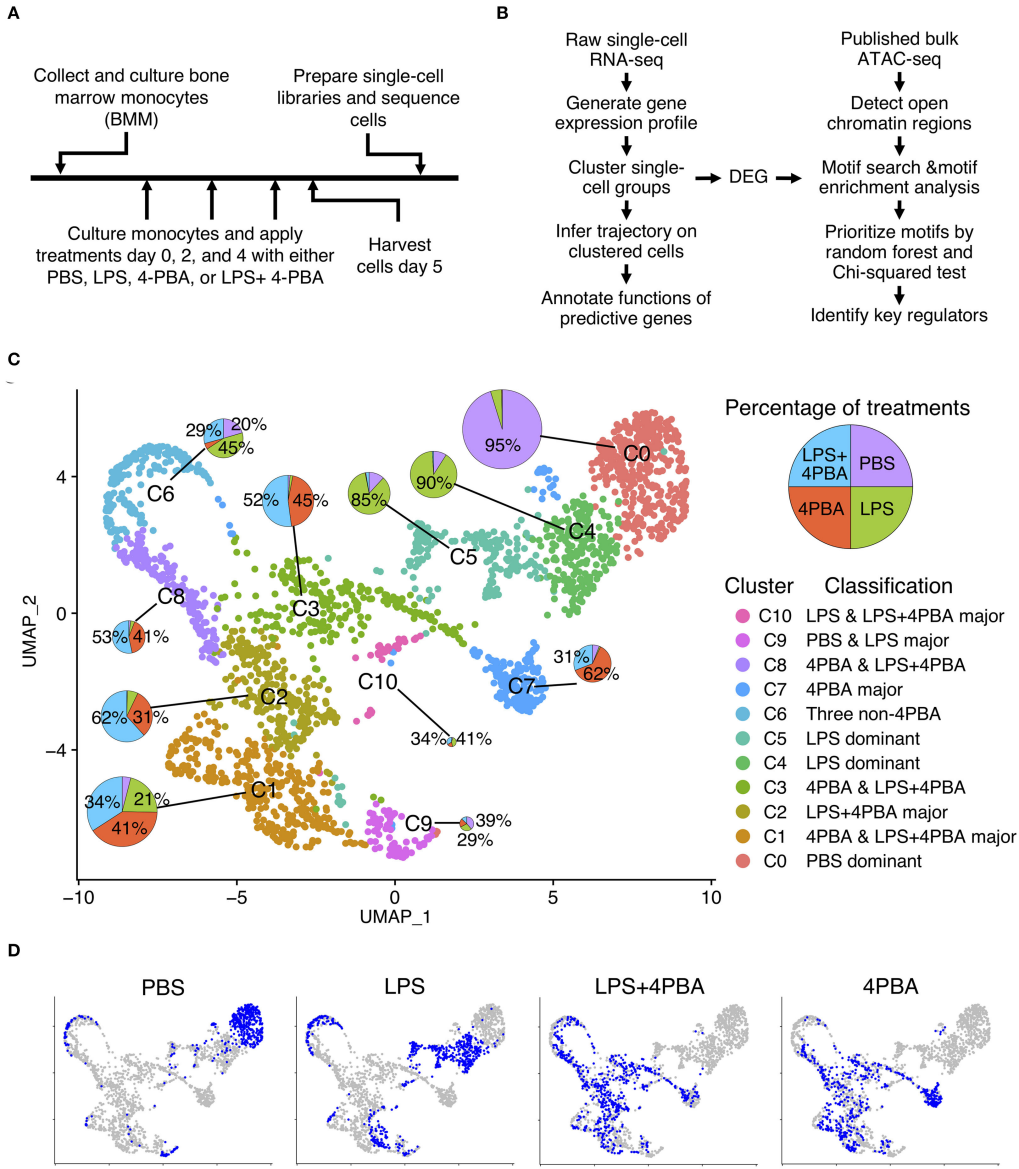
Single cell analysis of LPS and 4PBA treated monocytes. **(A)** Experimental design and data collection. **(B)** Data analysis pipeline. **(C)** Clustered cell groups. Dimension reduction using UMAP shows the diversity of cell populations in response to treatments. A pie chart denotes portions of treatments in a cluster. Sizes of the pie charts are correlated with the number of cells in each cluster. **(D)** Distribution of cells from each treatment or control samples. Total cells in a background are in grey color and cells in a sample are in blue color.

To stimulate chronic subclinical inflammation in monocytes, we isolated leukocytes from mouse bone marrow, cultured with monocyte selection media, and applied four treatments for 5 days (Figure 1A) following established protocol (5, 7, 32). Monocytes were persistently treated with super low dose lipopolysaccharide (LPS), in order to stimulate the chronic low-grade endotoxemia condition seen in patients (33–36). In order to investigate potential therapeutic functions of Sodium 4-phenylbutyrate (4PBA), we treated monocytes with 4PBA and LPS+4PBA, respectively. Phosphate-buffered saline (PBS) was used as a control. Harvested monocytes were prepared into single-cell libraries then sequenced using 10x genomics and Illumina platforms. We analyzed single-cell RNA-seq data using clustering analysis, trajectory analysis, and transcription factor binding motif enrichment in open chromatic regions to understand transitional states in monocytes (Figure 1B).

From single cell RNA-seq, we obtained a total of 225 million raw reads and 2,772 cells from four samples with 81 K of mean read pairs per cell and 67% sequencing saturation across samples (Supplementary Table 1). These parameters are higher than typically recommended values (50 K read pairs per cell and 30–50% of sequencing saturation). In addition, mean reads per gene per cell is 24 for our data (mean sequencing read pairs divided by the estimated number of cells and the median genes per cell, see Supplementary Table 1). This number is higher than many published datasets (37). These results suggest that our data can detect transcripts with low expression levels (38–40). We obtained gene expression profiles where 95.7% of sequenced reads had valid barcodes and 93.3% reads were successfully mapped to the reference genome. The overall summary statistics of the sequencing results indicated that sequencing depths were deep enough to identify cellular transition in monocytes.

We conducted quality control (QC) to filter cells based on percent of mitochondrial genes and the number of genes per cell (Supplementary Figure 1 and Supplementary Table 2). Average number of genes per cell across samples was 3,300 before QC and 3,700 after QC. Cells with too many and too few genes were removed to filter out outliers such as multiplets and dead cells. Among four samples, cells in PBS and 4PBA samples contained high percent of UMIs from mitochondrial genes before QC (PBS: 14.8% and 4PBA: 16.1%) but their percentage decreased after QC (PBS and 4PBA: 8.7%). Correlation value between the number of UMIs and the percent of mitochondrial genes reduced from −0.45 to −0.12 after QC, while correlation value between the number of UMIs and the number of genes remained the same (0.94). After the filtering procedure, a total of 14,035 genes were detected and 2,189 high quality cells were used for further analysis.

### scRNA-Seq Identified Activated Monocytes in Response to Subclinical Low-Dose Endotoxin, Which Were Suppressed by 4-PBA

We integrated the filtered individual expression profiles of monocytes treated with four treatments into one data set, and performed dimension reduction and clustering analysis. We identified 11 clusters (C0–C10) from ~2,200 monocyte cells from four treatments (Figure 1C and Supplementary Table 3). Interestingly, we observed that 11 clusters of monocytes from scRNA-seq consist of both homogenous and heterogeneous sub-populations in response to treatments. Among 11 clusters, three clusters are relatively homogenous clusters, which are dominated by cells originating from one sample. Other eight clusters are composed of cells from multiple samples. For example, almost all cells (95%) in cluster C0 are from the PBS treated sample, and cells in cluster C4 and C5 are mostly from the LPS treated sample (90 and 85%, respectively). Other clusters are consisted of cells from two or more treatments. For example, cells from cluster C2, C3, C7, and C8 are mainly cells treated by either 4PBA or LPS+4PBA. Cluster C1 has the second largest number of cells among all clusters, and these cells are mainly from three treatments (4PBA, LPS+4PBA, and LPS) with each contributes to more than 20% cells in cluster C1. Cluster C6 also has cells from three treatments (LPS, LPS+4PBA, PBS).

Distinct cell clusters with differentially expressed genes following the four different treatment regimens were shown in Figure 1D. Majority of PBS cells are found in cluster C0 (72%). Majority of LPS cells are separated into two clusters C4 (32%) and C5 (28%). UMAP plots of PBS and LPS treated samples have distinct distributions, respectively compared with other treatments with the exception of C6. In contrast, cells from LPS+4PBA and 4PBA are spread out into several clusters with more diverse expression patterns. The cells under these two treatments show substantial overlap in the dimension reduction plot, suggesting that majority of the effects of LPS are masked by 4PBA treatments, supporting a potential role of 4PBA in overriding the effect of LPS.

In summary, we found that C0 mainly includes cells from the control sample (PBS treatment). C4 and C5 are mainly LPS treated cells. The major populations in C2, C3, C7, and C8 are 4PBA and LPS+4PBA treated cells, which suggests that 4PBA masks the effect of LPS treatment in these clusters. C1 is a unique sub-population including cells from all three types of treatments but almost no cells from control samples. C6 and C9 are two smaller sub-populations where >90% of genes in C6 are expressed lower than in other clusters, and 95% of genes in C9 are expressed higher than in other clusters. C10 is a small cluster with only 41 cells. In contrast to the C0 cluster, where most control cells were located, cells in response to LPS, 4PBA, or LPS+4PBA are found in 10 clusters that are more heterogenous than the control samples in the dimension reduction plot, indicating that monocytes have heterogenous responses to LPS, 4PBA, or LPS+4PBA.

### scRNA-Seq Identified Two Distinct Clusters of Low-Grade Inflammatory Monocytes Primed by Subclinical Low Dose Endotoxin

**Figure 2:**
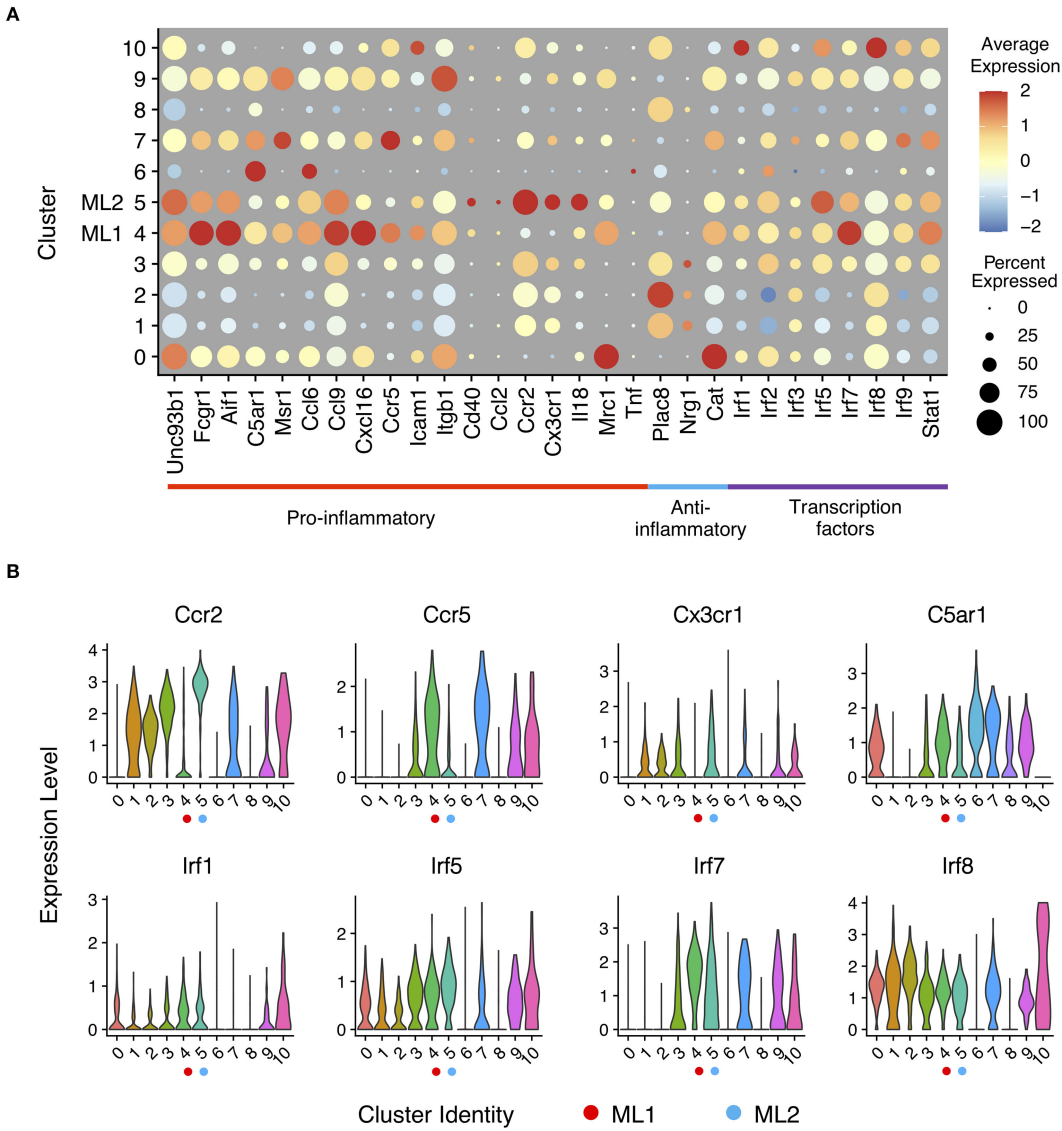
Expression profiles of inflammatory marker genes in monocytes response to LPS challenges. **(A)** Gene expression levels of pro- and anti-inflammatory marker genes and interferon-regulatory factor genes related to innate immune regulation in a dot plot. Sizes of dots are proportional to percentages of expressed cells in each cluster. **(B)** Relative expression levels of selected gene from **(A)** in violin plots showing expression levels of genes by clusters (bottom).

To better understand the functions of each cluster, we analyzed expression of known genes in each cluster using a dotplot. In a dot plot (Figure 2A), the average expression level of each gene in each cell cluster is represented by dot color, while the percentage of cells that each gene is expressed within each cluster is represented by the size of the dot. With single cell precision, our data confirms a well-reported phenomenon of monocyte priming by persistent challenge of super-low dose LPS, in contrast to the tolerance phenotype observed in monocytes challenged with higher dose LPS (6, 41). Our single cell analysis also confirmed previous reports showing limited expression of acute inflammatory cytokines such as Tnfα (42), indicating a low-grade inflammatory state induced by subclinical low dose LPS. Furthermore, we observed two distinct clusters of activated monocytes (cluster C4 and C5) following the persistent challenge with subclinical low dose LPS, which we termed as Ml1 (cluster C4) and Ml2 (cluster C5) that manifest unique biological features of activation. Consistent with previous reports with whole cell studies (32, 43), we observed that subclinical low dose LPS preferentially programmed monocytes into low-grade inflammatory states with elevated expression levels of chemokines (e.g., Ccl6, Ccl9, Cxcl16, etc.), chemokine receptors (e.g., Ccr2, Ccr5, Cx3cr1), scavenger receptors and adhesion molecules (e.g., Msr1, Fcgr1, Icam1), and co-activators (e.g., Aif1 and Cd40) (Figure 2 and Supplementary Figure 2, see Ml1 and Ml2 clusters).

Ml1 cells preferentially express C5ar1, encoding the complement receptor protein CD88; multiple interferon activated genes (e.g., ifit2, ifit3 etc.); Msr1 encoding scavenger receptor R1 (SR-A1); as well as Ccr5 encoding a key chemokine receptor involved in the recruitment of innate monocytes to inflamed tissues (44). On the other hand, the Ml2 have preferential expression of Ccr2 (Figure 2B), another key chemokine receptor involved in the recruitment of inflammatory monocytes; Cx3cr1, a signature chemokine receptor identified on classically activated monocytes from both murine and human peripheral blood cells (44, 45). However, the levels of C5ar1 were relatively low on Ml2 cells. Our data were also consistent with previous studies showing that human intermediate monocytes tend to express higher levels of CCR5, while the classical monocytes tend to have higher levels of CCR2 (6). Although both sets may be involved in the pathogenesis of inflammatory diseases such as atherosclerosis, the differential expression of distinct inflammatory gene sets revealed from our study suggests their unique contributions. The Ml1 cells resemble the intermediate inflammatory monocytes coordinating immune-enhancing inflammatory responses identified from human patients with auto-immune diseases such as rheumatoid arthritis (RA) as well as atherosclerosis (46–48). Ml2 cells may represent classically activated inflammatory monocytes being recruited to inflammatory sites such as atherosclerotic plaques (49–51).

Given the previous findings that low-grade inflammatory monocytes have elevated transcription factors such as IRF5 (6, 32), we examined the levels of interferon-regulatory factor family transcription factors (e.g., Irf1, Irf2, Irf3, Irf5, Irf7, Irf8, and Irf9) among the sub-populations, represented by the dot plot as shown (Figure 2A). Indeed, we found that the expression of pro-inflammatory Irfs such as Irf1, Irf5, Irf7, and Irf9 were all elevated in Ml1 and Ml2 cells as compared to PBS control cluster C0. On the other hand, the levels of Irf3 and Irf8 were largely not impacted by LPS challenge as compared to PBS control. Interestingly, our cluster analysis revealed that the Ml1 cells had higher levels of Irf1 and Irf7 (Figure 2B) as compared to the Ml2 cells. In contrast, Ml2 cells had relatively higher levels of Irf5 (Figure 2B) as compared to Ml1 cells, suggesting potentially distinct activation mechanisms among these two distinct clusters. Together, our data reveal two distinct sub-population of low-grade inflammatory monocytes with distinct inflammatory features programmed by subclinical low-dose LPS, potentially governed by unique Irf transcription factors.

### 4-PBA Potently Reprograms an Anti-Inflammatory Monocyte Phenotype and Masks the Effects of Subclinical Low Dose LPS

4-phenylbutyric acid (4-PBA) is a potent chemical compound capable of relieving cellular stress, and has been shown to exhibit beneficial anti-inflammatory effects both *in vitro* and *in vivo* (52–54). To test its efficacy in reprogramming monocytes, we cultured murine monocytes with 4-PBA for 5 days as we previously described (9). As shown in Figure 2, 4-PBA treatment selectively programmed three clusters of anti-inflammatory cells (cluster C1, C2, and C3), with reduced expression of C5ar1, Msr1, Ccl6, Ccl9, Cxcl16, Ccr5, Icam1, and Cd40 as compared to PBS treated cluster C0 or LPS treated clusters C4 and C5 (Figure 2A). In contrast, 4-PBA drastically induced the expression of selected anti-inflammatory mediators such as Plac8 in clusters C1, C2, and C3. 4-PBA treated cells also had reduced levels of transcription factors Irf1 and Irf5, consistent with a reduced inflammatory state (Figure 2A). Our results also revealed some intriguing phenomena in terms of the expression of catalase (gene name cat), which was reduced by both 4-PBA and subclinical low dose LPS. Surveying the populations detected with both 4-PBA and LPS challenge, we found that 4-PBA largely masked the effects of LPS as shown in clusters C8 and C9. Our data revealed that indeed 4-PBA preferentially suppressed the polarization of both subsets of low-grade inflammatory monocytes, consistent with its anti-inflammatory effects with animal models (53, 55, 56).

**Figure 3:**
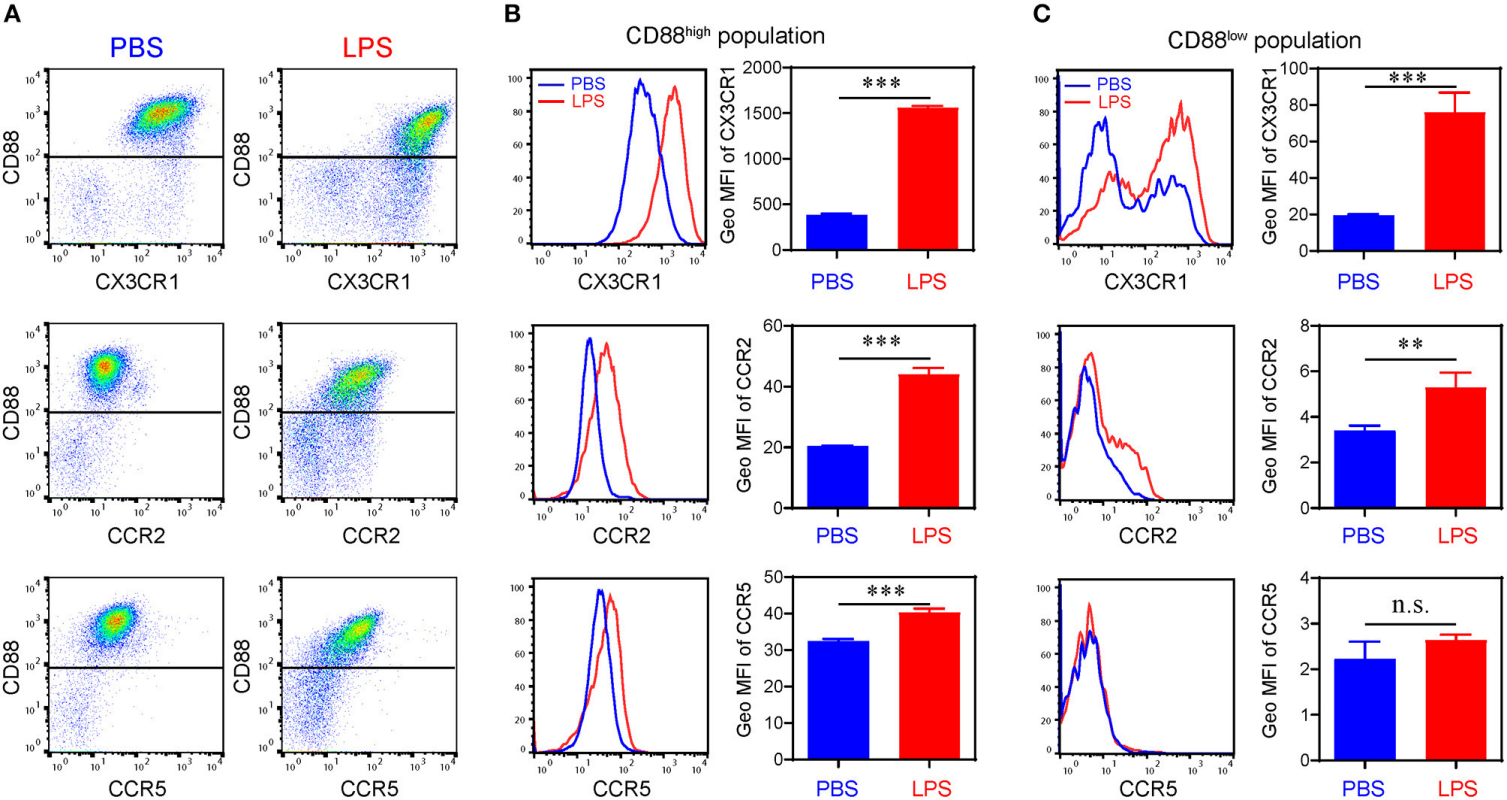
Differential expression of chemokine receptors on bone marrow derived monocytes. **(A)** Representative flow cytometry data of the surface levels of CD88, CX3CR1, CCR2, and CCR5 on monocytes primed with PBS or super low dose LPS (100 pg/ml) for 5 d. **(B)** Representative histograms (left panels) and quantification of geometric mean fluorescence intensity (right panels) demonstrating CX3CR1, CCR2, and CCR5 expression on CD88^high^ population. **(C)** Representative histograms (left panels) and quantification of geometric mean fluorescence intensity (right panels) demonstrating CX3CR1, CCR2, and CCR5 expression on CD88^low^ population. n.s. not significant; **P < 0.01; ***P < 0.001. Student's t-test.

We further confirmed the induction of key representative protein molecules through flow analyses (Figure 3). Consistent with our single cell sequencing analysis, monocytes challenged with subclinical low dose LPS are separated through flow analysis into at least two sub-population based on CD88 (C5ar1) expression. Persistent challenge with subclinical low dose LPS also induced the surface expression of CCR2, CCR5, and CX3CR1. Interestingly, although the induction of CCR2, CCR5, and CX3CR1 occurred on the CD88 high population, CCR5 was primarily induced on the CD88 high, but not the CD88 low population (Figure 3C). These results in protein expression levels are consistent with gene expression level in the scRNA-seq data. Although the absolute number of cells in the two subpopulations is different from the results in scRNA-seq, such difference might be explained by the differences in transcription levels and protein levels.

### Potential Ontogeny of the Low-Grade Inflammatory Monocyte Clusters With Trajectory Analysis

Given the clear advantage of single cell analysis in defining the heterogeneity of cells, one may infer the ontogeny of activated cell populations with the pseudo-time trajectory analysis. To understand the transition of cells between different states, we performed trajectory analysis on the scRNA-seq data. The cluster C0 served as the natural origin of the trajectory, because it is PBS-treated control. A starting cell was randomly selected from the PBS sample as the root of pseudo time in PBS dominant cluster C0. To choose the most appropriate clustering method, we used Dynbenchmark, which compared and recommended trajectory methods based on computing capacity and prior information such as the choice of the starting cell (16). After testing several methods suggested by Dynbenchmark, we have selected minimum spanning tree (MST) method to infer a pseudo-time trajectory because the trajectory best fit the observed subpopulations. The MST method used mclust (17) to find the clustering groups and to generate MST based on distances between centers of groups as weights. Using mclust, 17 subgroups were clustered as the best fit to our data and centers of these subgroups were determined as milestones. Based on distances of path branches where is a path between two milestones, the minimum weights were decided and MST was calculated on the UMAP coordinates.

**Figure 4:**
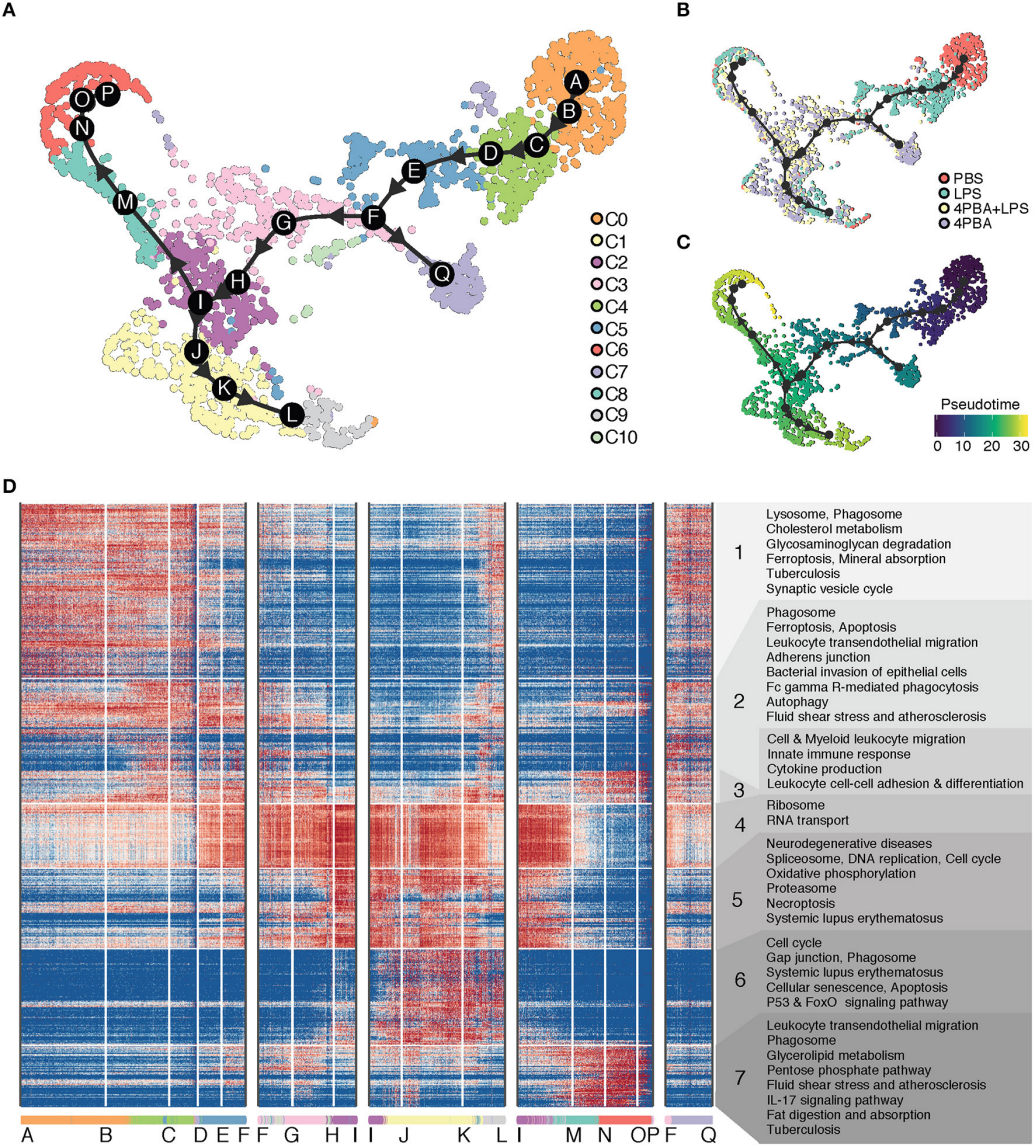
Trajectory analysis of scRNA-seq data. **(A)** Inferred trajectory of cellular transitions along 17 milestones (A–Q) on the clustering map. There are five trajectory branches: milestones A-to-F; F-to-I; I-to-L; I-to-P; and F-to-Q. A legend on the right denotes 11 clusters (C0–C10) with different color codes. **(B)** Inferred trajectory on the sample map with control and treatments. A legend on the right denotes four samples (PBS, LPS, LPS+4PBA, 4PBA) with different color codes. **(C)** Inferred trajectory on a pseudo time map. A starting cell was defined from a cell treated with PBS and in C0. **(D)** Gene expression profile and enriched functional annotation of 936 predictive genes that change expression level along trajectory branches. The predictive genes were clustered and visualized in a heatmap. The heatmap was split into 7 classes with functional terms on the right side. Cells from 11 clusters are sorted along the trajectory and 17 milestones are marked on the bottom of the heatmap.

We inferred a trajectory with 17 milestones (A to Q) that reflect the progression of immune responses in monocytes in response to LPS and 4PBA challenges (Figure 4). The main transition of cellular states on the trajectory is from a naïve state (PBS treatment, cluster C0) to pro-inflammatory state (LPS treatment, cluster C4 and C5) and to anti-inflammatory state (4PBA treatment, cluster C3, C2, C1) (Figures 4A,B). We broke the trajectory into five branches based on the biological information and expression coherency. First branch of interest is the trajectory from A to F. This branch represents the response of monocytes to LPS treatment (Figure 4B) and accounts for 1/3 of pseudo-time scale in the UMAP plot (Figure 4C). From the milestone F, two branches were derived; the trajectory from F to Q and the trajectory F to I. Second branch of interest is the F-to-I branch, which represents the transition from LPS treated state (pro-inflammatory) into 4PBA+LPS treated state (anti-inflammatory). Two branches were spread out from this F-to-I branch, including I-to-P branch and I-to-L branch. The I-to-P branch represents the transition into cluster C8 and C6, and the I-to-L branch represents the transition into a large cluster C1 and then a minor cluster C9.

To understand the changes in the gene expression patterns and the gene functions along these branches, we identified genes highly expressed in each branch, visualized their expression levels, and performed functional enrichment analysis of these genes in KEGG pathway terms. We divided highly expressed genes into seven classes based on expression patterns (Figure 4D). Note that these classes of genes are essentially clusters of genes and are different from cell clusters. Genes in class 1 are highly expressed in the beginning of A-to-F branch where cells in cluster C0 are sorted in the beginning of the branch, and the gene expression of class 1 is gradually decreased along A-to-F branch which marks progression of the pro-inflammatory responses. Genes in class 1 are enriched with function in lysosome and phagosome, which are relevant to key monocyte functions such as phagocytosis (57, 58). In contrast, class 4 includes genes involved in ribosome and RNA transport. The levels of these genes in class 4 were low in the beginning of A-to-F branch which includes pro-inflammatory cells, then gradually increased through F-to-I branch and coincided with the reduced expression of pro-inflammatory genes in class 1 and class 2. This trend continues through I-to-L branch, and decreases through I-to-P branch. Class 5 includes genes from spliceosome and proteasome as well as oxidative phosphorylation. The distribution patterns of class 5 were similar as compared to class 4, but the activation of these genes was more concentrated on milestone I and cells in cluster C2 toward the end of F-to-I branch and continued toward I-to-L branch. Increased oxidative phosphorylation in monocytes is associated with the anti-inflammatory polarization. This is consistent with the fact that these cell clusters within the F-to-I branch as well as I-to-L branch are 4-PBA responsive clusters exhibiting anti-inflammatory phenotypes and reduced expression of pro-inflammatory genes.

Class 6 and class 7 include genes activated on two opposite branches (I-to-L and I-to-P branches), respectively, which suggest two distinctly polarized states in cells challenged with 4-PBA together with LPS. While the I-to-L branch correlated with anti-inflammatory features as well as enhanced expression of genes involved in oxidative phosphorylation, the I-to-P branch preferentially expressed genes involved in glucose metabolism such as the PPP pathway as well as genes involved in lipid digestion and absorption. Our data suggest that the effects of LPS and 4-PBA may be competitive in shaping the final outcome of monocyte fates, with the effects of 4-PBA mostly taking the upper hand among the I-to-L branch while the effects of LPS taking a slight dominance in the I-to-P branch. Further functional confirmation will be needed to examine the detailed mechanisms.

### Machine Learning Predicts Cluster-Specific Motifs With Better Performances Than Enrichment Analysis

**Figure 5:**
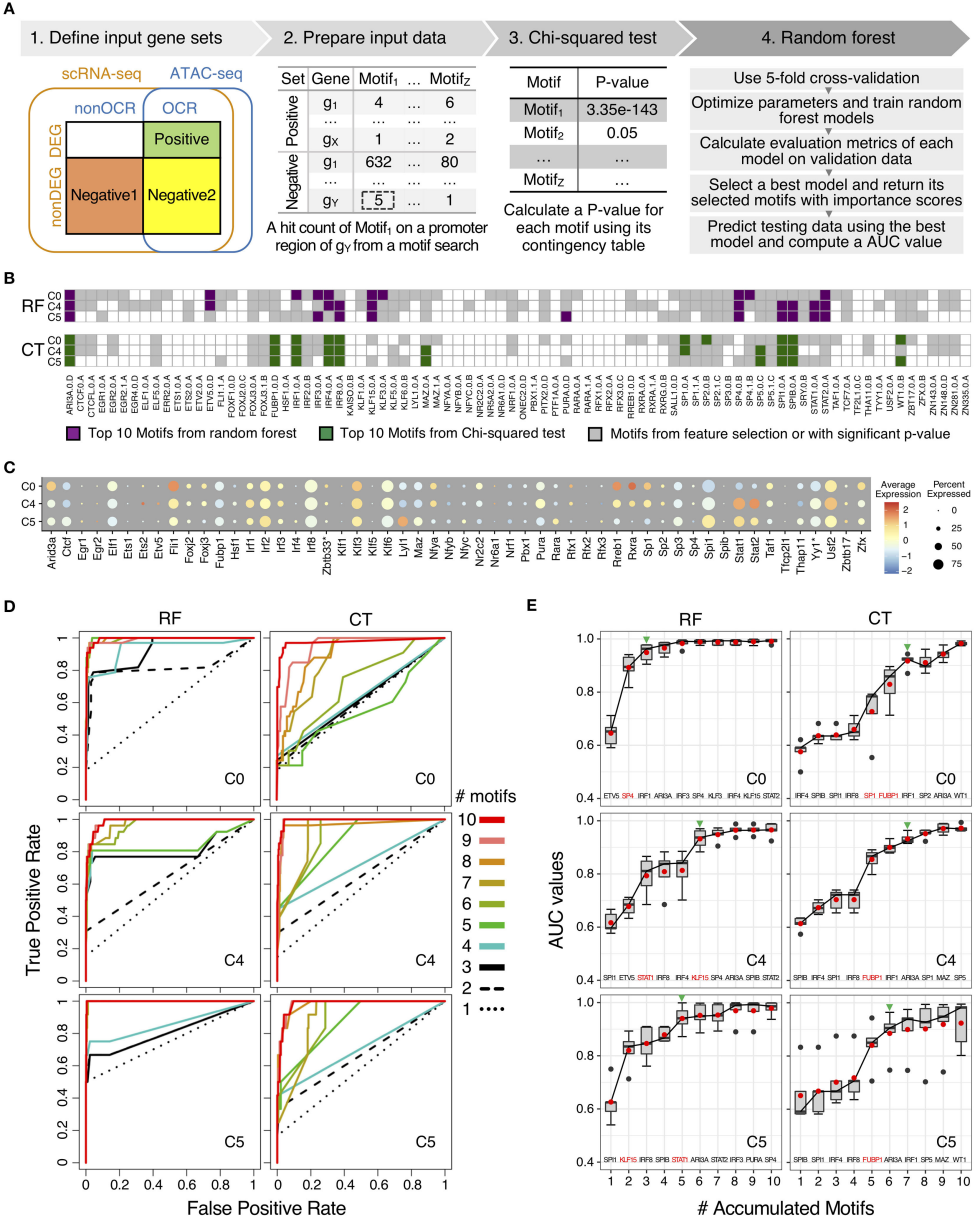
Comparison of motifs enrichment analysis and feature selection by random forest (RF) and Chi-squared test (CT). **(A)** Pipeline of motif analysis composed of four steps including preparation of input data sets based on positive and negative gene sets and performing Chi-squared test and random forest. **(B)** Motif selection results of cluster C0, C4, and C5 by RF and CT. Total 84 selected motifs were visualized in tile plots. **(C)** Expression profile of transcription factor genes corresponding to the selected motifs in cluster C0, C4, and C5. Some motifs have different names for their genes: KAISO—Zbtb33; TF2L1—Tfcp2l1; THA11—Thap11; TYY'—Yy1; ZBT17—Zbtb17. **(D)** Prediction performance of cluster C0, C4, and C5 with top N motifs in auROCs by RF and CT. **(E)** Prediction performance of cluster C0, C4, and C5 with top N motifs in auROC values from 5-fold cross-validation by RF and CT. Green arrowheads indicate the number of accumulated motifs where average median and mean auROC values are equal to or higher than 0.9. Names of top 10 motifs were listed on the bottom of the box plot, and one or two motifs with the highest auROC improvement were highlighted in red color.

To further elucidate the regulatory mechanisms of scRNA-seq results, we used a machine learning method to identify and to prioritize potential key transcription factors using binding motifs and mouse monocyte ATAC-seq data and scRNA-seq. In particular, we have adapted a guided, regularized random forest method using feature selection to prioritize motifs that are enriched in the proximal regulatory regions and on open chromatin regions of genes in each cluster. As compared our previous method using logistic regression with L1 regularization (59), random forest method allows us to explore different approach to combine features in the feature selection process. The results from machine learning method was compared with the results from conventional Chi-squared test to determine which method has a better performance based on auROC and auPRC curves (Figure 5A).

For motif enrichment analysis, we performed Chi-squared test to identify enriched motifs and random forest to select important motifs using feature selection (Figure 5A and Supplementary Figure 3). As input data, positive gene sets and negative gene sets were defined for individual cluster, respectively according to gene expression levels and chromatin accessibility of transcription factors binding sites of the target genes (Supplementary Figure 2 and Supplementary Table 4). Genes in positive sets were differentially expressed genes (DEG) with open chromatin regions (OCR) that were annotated as promoter-TSS regions by HOMER (26). For negative gene sets, we tested two approaches, including negative gene set 1 and negative gene set 2. Genes in both negative gene sets were non-DEGs, but genes in negative gene set 1 do not have promoter-TSS open chromatin regions (non-OCR) and genes in negative gene set 2 have OCR. Number of genes in positive and negative sets were shown in Supplementary Table 4. With genes in positive and negative gene sets for individual clusters, we next searched known motifs on the promoter-TSS regions of genes in positive and negative gene sets for individual clusters. We counted motif hits per gene in the OCR regions and generated hit count profiles as input data of further analysis. Since genes in negative set 1 do not have OCRs, we searched motifs in 1,200 bp sequences up- and down-stream of TSS for these genes. We used 1,200 bp because this is the average length of annotated open chromatin regions in promoter-TSS regions. We performed motif enrichment analysis with motifs in positive and negative gene sets by using Chi-squared test and random forest (Figure 5A). We compared performances of motif enrichment analysis between to input gene sets with negative gene set 1 and negative gene set 2 in each cluster with auROC curves (Supplementary Figure 4). Average mean AUC values across 11 cluster of gene set 1 is 0.986 and those of gene set 2 is 0.691 (Supplementary Figure 4A). For CT, average –log_10_ *p*-value of gene set 1 is 21.4 and those of gene set 2 is 1.9 (Supplementary Figure 4B). Since negative gene set 1 showed better results from both RF and Ct, we used gene set 1 for further analysis.

We scanned 531 mouse motifs and we detected 84 motifs that are enriched in one or more clusters (Figure 5B and Supplementary Figure 5). Among these motifs, 59 motifs were detected using Chi-squared test (CT), whereas RF identified 82 motifs. In order to prioritize potential key regulators in low-LPS inflammation in monocytes, we perform features selection by selected top 10 motifs by importance scores from RF and by *p*-value from CT (Supplementary Table 5). We identified top 10 motifs for each of the 11 clusters and because there are motifs that are in the top 10 list for more than one clusters, we obtained 33 unique motifs in total, with 30 motifs from RF and 16 motifs from CT. There are 13 motifs were identified as top 10 motifs for both RF and CT methods, and 3 motifs and 16 motifs were unique in CT and in RF, respectively.

Moreover, the distribution of top 10 motifs across 11 cluster is distinct between RF and CT (Figure 5B and Supplementary Figure 5). We found that top 10 motifs from CT are mostly the same across different clusters, whereas top 10 motifs from RF are very different in each cluster (Supplementary Figure 5A). For example, both CT and RF identified five IRFs (IRF1/2/3/4/8) as important motifs. However, using CT, IRF1/4/8 were identified as top 10 motifs across 8 or more clusters. In contrast, using RF, no IRF were considered as top 10 across more than 6 clusters. In particular, IRF1 was identified as important motifs in all 11 clusters by CT, but these clusters have very different biological functions. In contrast, RF defines IRF1 as a common top motif in cluster C0, C1, C2, and C3, but not in C4 or C5. Cluster C0–C3 all have naïve or anti-inflammatory characteristics, where as C4 and C5 are inflammatory clusters. These results suggest that RF selected motifs that are more cluster-specific than CT.

To investigate relationship between top 10 motifs in RF and CT, we looked into the patterns of the motif sequences (Supplementary Table 6). We grouped top 10 motifs by transcription factor family, and there are 33 unique motifs found in at least one in 11 clusters from both CT and RF. These 33 unique motifs belong to 10 TF families. We found that RF usually has more motifs for each TF family than CT. For example, as three-zinc finger Krüppel-related factors, RF selected 8 motifs (KLF15.0.A, KLF3.0.A, SP1.0.A, SP1.1.A, SP3.0.B, SP4.0.B, SP4.1.B, SP5.0.C) and CT selected 3 motifs (SP1.0.A, SP2.0.B, SP5.0.C). This result suggest that RF is more sensitive and can pick up small difference between highly similar motifs from the same gene family.

We also analyzed the gene expression levels of transcription factors of 84 motifs to understand gene regulation by TFs together with motif enrichment analysis results (Figure 5C and Supplementary Figure 5B). Since some TFs have two motifs, there were 75 genes and 84 motifs. Among these 75 genes, only 64 genes were detected in scRNA-seq data. The fold change of expression of these 64 genes are not as high as compared to other marker genes (Figure 2A). Also, expression levels of TFs do not show clear association with motif enrichment results when we compared individual cluster along the pseudo-time. Some TFs, for example, IRF4 and SPIB are not highly expressed, but their motifs were selected as top candidate motifs by both RF and CT. In other situations, for example, IRF8 and SPI1, highly expressed genes do have motifs that are also selected. Finally, genes such as STAT1 and STAT2 are both highly expressed, but their motifs were only selected by RF but not CT. This result suggests that transcription levels and protein levels may not be correlated for some TFs but for others, there is correlations between the expression level and motif enrichment.

To test the performance of top 10 motifs for RF and CT, we generated ROC curves for these motifs and analyzed how ROC curves changes for each method. First, both CT and RF show excellent auROC values with top 10 motifs. Average AUC across 11 clusters with all 84 motifs from RF was 0.986. Using only top 10 motifs from RF, we obtained an AUC of 0.979 and using top 10 motifs from CT, we obtained an AUC of 0.961. These performances indicated that only with top 10 motifs from both methods can effectively classify positive and negative gene sets.

Since both approaches provide high auROC values with top 10 motifs, we then evaluate degree of separability with top 10 motifs by comparing performance of motif selection of each method by predicting testing data with top N motifs (*N* = 1–10) by including motifs one by one (Figures 5D,E and Supplementary Figures 6, 7). We trained random forest models with top 10 selected motifs from CT and RF, respectively. When we applied the models to testing data, we used top N motif to evaluate the prediction performance. By increasing the number of motifs, ROC curves approached to the top-left corner, indicating the perfect classification shape with higher true positive rate and lower false positive rate (Figure 5D and Supplementary Figure 6). When we compared ROC curves between RF and CT, ROC curves with RF motifs reached the better classification results with fewer motifs consistently. The same result was found for auPRC (Supplementary Figure 6).

For example, in auROC graphs of cluster C0, ROC curves of using up to five motifs by CT were near the diagonal line (Figure 5D), with average auROC below 0.8 at 5 motifs. In contrast, the ROC of top 1 motif by RF was near the diagonal line and showed an average auROC of 0.6. When the second-best motif was added, the average auROC is already higher than 0.9 (Figure 5E and Supplementary Figure 7A). As mentioned before, average AUC values cross 11 clusters with top 10 motifs by both methods were high and close to one. However, motifs selected by RF using importance score approach high AUC values with fewer motifs. For example, in cluster C0, seven motifs selected by CT method were needed to reach AUC = 0.9, but only three motifs from RF were required for the same performance. This difference is observed in other clusters consistently (Figure 5E). In summary, these results indicate that motifs provided by RF show better performances with fewer number of motifs.

To understand these differences between RF and CT, we checked the number of target genes of top 10 motifs in positive gene sets by RF and CT (Supplementary Figure 7B). The total number of target genes that could be potentially regulated by transcription factors (TF) of top 10 motifs were similar between RF and CT methods. However, as we included top motifs one by one, the accumulated number of target genes with RF motifs increased faster. For example, two or three motifs by RF covered more than 70% target genes in positive gene sets, while CT needed 5 motifs in average. This suggests that RF provides higher importance scores to motifs that are highly connected to target genes. Based on these prediction performances as well as the gain of target genes, we conclude that selected feature by RF method are more specific for each cluster with higher degree of separability than feature selection by CT method.

## Discussion

Monocytes are innate immune cells that play crucial and diverse roles during the modulation of host inflammatory environment. A better understanding with regard to the monocyte activation dynamics is needed in order to guide the future effective treatments of both acute and chronic inflammatory disorders. Previous study with monocytes/macrophages by bulk RNA-seq data allows the identification of key differentially expressed mediators for further analysis based on their average expression levels from the whole cell population (7). However, the bulk RNA-seq approach will not be able to properly differentiate diverging sub-populations of monocytes differentially activated toward multiple trajectories. In this project, we performed single-cell RNA-seq analyses of monocytes that allows us to quantify gene expression levels for individual monocytes, overcoming the limitation associated with the conventional bulk RNA-seq.

Our single cell analysis revealed two distinct populations of activated monocytes (C4 and C5) when persistently challenged with subclinical super-low dose LPS, which we named as Ml1 and Ml2. Consistent with the previous studies with the conventional approaches, we confirmed that indeed the persistent challenge with SLD-LPS preferentially induced the low-grade activation of monocytes as reflected in the increased transcription factor signatures of IRF1, 5, and 7 (6, 32, 60, 61) in the LPS responsive clusters of C4 and C5. Both IRF1 and IRF5 were shown by conventional biochemical approaches as the key transcription factors involved in the polarization of inflammatory monocytes (62, 63). Furthermore, LPS-activated monocyte clusters (C4, C5) also express elevated levels of chemokines such as Ccl2, Ccl6, Ccl9, and Cxcl16, adhesion molecule Icam1, and phagocytic receptor Fcgr1 (Figure 2). Our single cell analyses also revealed unique features of two distinct clusters activated by SLD-LPS. Ml1 cells in cluster 4 preferentially expressed C5ar1, Ccr5 and resembled the intermediate inflammatory monocytes observed in both human and mice systems (6, 58). In contrast, Ml2 cells in cluster 5 preferentially expressed Ccr2, Cx3cr1, and Il18, resembling the classical Ly6C^hi^ inflammatory monocytes observed previously (58, 64). Ml1 monocytes might be preferentially associated with propagating low-grade inflammatory processes, while Ml2 monocytes might be implicated in coordinating additional pathophysiological events such as patrolling, adherence, phagocytosis through limited proliferation. Given the powerful approach of single cell sequencing analysis that provides additional details of cellular activation states, similar approaches can be applied in the future to define additional states of monocyte programming during the pathogenesis of wide arrays of inflammatory diseases such as chronic atherosclerosis and acute sepsis. For example, different subsets of low-grade inflammatory monocytes might be differentially involved in the initiation of low-grade inflammation and the accumulation of foamy macrophages. On the other hand, prolonged challenges with higher dose LPS may lead to dysfunctional innate leukocytes with an exhausted phenotype characterized by pathogenic inflammation and immuno-suppression associated with elevated sepsis (65). Future single cell sequencing analysis will be helpful in defining additional sub-populations of monocytes adopting distinct phenotypic states representing both chronic atherosclerosis and acute sepsis, dependent upon the duration and intensity of external danger signals.

In contrast, 4-PBA treated cell clusters (C1, 2) had reduced expression of these inflammatory genes, consistent with the anti-inflammatory effects of 4-PBA reported previously (7, 66). When challenged together, the effects of 4-PBA can largely over-shadow the polarizing effects of super-low dose LPS. However, perhaps due to the competing nature of 4-PBA and super-low dose LPS, cells co-treated with LPS and 4-PBA also give rise to two distinct populations with unique trajectories. It is interesting to note that one of the populations derived from LPS plus 4-PBA treatment expressed genes enriched with metabolic PPP (pentose phosphate pathway), while the other branch exhibited genes involved in oxidative phosphorylation (Figure 4). Our findings are consistent with emerging studies revealing a close connection between metabolic alterations with monocyte polarization (67). Both the PPP and oxidative phosphorylation processes have been implicated in maintaining monocyte homeostasis, with PPP pathway generating NADPH involved in anti-oxidative processes and the oxidative phosphorylation pathway leading to enhanced ATP generation (67, 68). Future mechanistic studies are needed to further define the homeostatic effects of 4-PBA in preferentially re-programing monocytes *in vitro* and *in vivo*.

Gene expression profile at the single-cell level can be used to infer cellular changes on the pseudo-time course from samples with one time point. In particular, this study treated monocytes with four treatments for 5 days to induce the low-grade and non-resolving inflammation *in vitro*. Based on results from trajectory analysis, we predicted cellular states of monocytes in response to inflection by LPS and anti-inflammatory treatment by 4PBA from naïve status by PBS. We have tested several trajectory finding algorithms and surprisingly, the best methods established by published benchmarking experiment performed poorly in our data set. The predicted trajectories either do not accurately follow the observed changes in the cell population or, in some cases, fall outside of the observed cell populations. We have found that the key step to determine a visually optimal trajectory is to fine tune the number of mile stone cells and number of clusters in the data. Determining number of clusters has been a major challenge in the field of unsupervised clustering analysis. More robust approaches would be needed to automate the process of trajectory analysis in the future.

To identify regulatory genes that modulate cellular states and underlying mechanisms of responses, we integrated scRNA-seq results with bulk ATAC-seq data from monocytes. Relying on scRNA-seq alone is limited to determine regulators that modulate the expression, since transcriptome data is designed to capture transcripts of responsive genes not transcription factors or regulatory RNAs that are already present in cells or not detected by the technology. Using the published ATAC-seq data from the cell type, monocyte, supplemented the missing information of participating transcription factors with the accessible chromatin regions where transcription factors can bind to in monocytes. By integrating two types of data sets, we predicted potential transcription factors that regulate differentially expressed genes in each group of cells. From 530 motifs in HOCOMOCO mouse DB, our RF method selected 10 motifs for each cluster and achieved high, average auROC of 0.96, suggesting that these motifs and their associated TFs can be key regulators controlling gene expression of monocytes.

We choose random forest method because it has been demonstrated to have good performances for gene expression data analysis in several papers (59, 69, 70). We also tested another methods using Lasso and Elastic-Net Regularized Generalized Linear Models (71) in our preliminary analysis. However, the performance is not comparable to the random forest method and this model is not included in our final result. Additionally, we found that random forest approach can always achieve similar performance with fewer motifs selected as compared to motif enrichment-based approach. This highlights the usefulness of machine learning methods in detecting motif combination through optimization across multiple motifs in contrast to enrichment test where one motif is considered at a time (72). The top 3 motifs selected by random forest can achieve a high auROC of 0.90 for some clusters, which provides a substantial reduction of the search space from 530 motifs to only 3 motifs, which is highly useful for determining candidate regulators from RNA-seq data. Finally, RF identified genes such as STAT1 to be key regulatory genes related to newly discovered Ml1 and Ml2 sub-populations of monocytes but not in the homeostasis population of monocytes. This result is not obtained by enrichment test. With the known function of STAT1 in polarizing inflammatory monocytes (73), our results suggest that the RF method implemented in this analysis provides biologically relevant prediction than traditional approaches. Additional experimental validations will be needed in future works to validate the predicted motif enrichment by perturbing the TFs with such binding sites.

## Data Availability Statement

The single-cell RNA-seq data generated in this study are publicly available in the Gene Expression Omnibus under accession number GSE160450 (https://www.ncbi.nlm.nih.gov/geo/query/acc.cgi?acc=GSE160450).

## Ethics Statement

The animal study was reviewed and approved by The Virginia Tech Institutional Animal Care and Use Committee.

## Author Contributions

LL and SL conceived the experiment. SG performed the experiments and generated data. JL analyzed the data and generated the figures and tables. All authors contributed to manuscript writing.

## Conflict of Interest

The authors declare that the research was conducted in the absence of any commercial or financial relationships that could be construed as a potential conflict of interest.
