# Supplementary material for: Single Cell RNA-Seq and Machine Learning Reveal Novel Subpopulations in Low-Grade Inflammatory Monocytes With Unique Regulatory Circuits
Source: Front Immunol. 2021 Feb 23;12:627036. doi: 10.3389/fimmu.2021.627036 (PMC7940189; doi:10.3389/fimmu.2021.627036)
Supplement: Supplementary file 1 [file Data_Sheet_1.docx]

SUPPLEMENTARY TABLE AND FIGURES

**Table S1.** Summary statistics of monocyte single-cell RNA-seq.

| **Sample** | **Key Summary** | | | | **Sequencing** | | **Mapping** |
| --- | --- | --- | --- | --- | --- | --- | --- |
|  | Estimated number of cells | Mean read pairs per cell | Median genes per cell | Sequencing saturation | Number of read pairs | Valid barcodes | Reads mapped to genome |
| **PBS** | 672 | 79,432 | 3,251 | 67.3% | 53,378,770 | 95.60% | 92.80% |
| **LPS** | 666 | 83,892 | 3,740 | 65.4% | 55,872,530 | 95.80% | 93.30% |
| **LPS4PBA** | 751 | 79,543 | 3,404 | 65.4% | 59,736,809 | 95.70% | 93.70% |
| **4PBA** | 683 | 81,829 | 3,342 | 68.9% | 55,889,689 | 95.80% | 93.30% |
| **Average**  **(Total)** | 693  (2,772) | 81,174 | 3,434 | 66.8% | 56,219,450  (224,877,798) | 95.7% | 93.3% |

**Table S2.** Summary statistics of quality control metrics.

|  | **Metrics** | | **4PBA** | **LPS** | **LPS4PBA** | **PBS** | **Total** |
| --- | --- | --- | --- | --- | --- | --- | --- |
| **Default filtering during loading step** | Total number of genes | | 13,144 | 12,467 | 13,431 | 12,177 | 14,035 |
|  | Total number of cells | | 666 | 653 | 735 | 645 | 2,699 |
|  | Average number of genes per cell | | 3,228 | 3,440 | 3,340 | 3,116 | 3,283 |
|  |  | Minimum | 201 | 207 | 226 | 208 | 201 |
|  |  | 1st quartile | 1,873 | 2,206 | 1,924 | 1,897 | 1,952 |
|  |  | Median | 3,394 | 3,761 | 3,486 | 3,334 | 3,494 |
|  |  | 3rd quartile | 4,362 | 4,558 | 4,617 | 4,244 | 4,436 |
|  |  | Maximum | 7,578 | 7,018 | 7,344 | 7,037 | 7,578 |
|  | Average number of reads per cell | | 14,028 | 16,809 | 15,698 | 13,447 | 15,016 |
|  |  | Minimum | 522 | 516 | 507 | 512 | 507 |
|  |  | 1st quartile | 5,253 | 7,761 | 6,368 | 5,973 | 6,347 |
|  |  | Median | 12,211 | 15,816 | 13,675 | 12,226 | 13,406 |
|  |  | 3rd quartile | 18,736 | 22,798 | 21,643 | 18,860 | 20,998 |
|  |  | Maximum | 64,142 | 65,764 | 59,189 | 66,436 | 66,436 |
|  | Average percent of mitochondria genes per cell | | 16.0986 | 8.587 | 13.44 | 14.7744 | 13.241 |
|  |  | Minimum | 0.1506 | 0 | 0 | 0.0738 | 0 |
|  |  | 1st quartile | 6.8681 | 4.085 | 5.87 | 6.2657 | 5.7 |
|  |  | Median | 9.0391 | 5.791 | 7.661 | 9.6603 | 7.764 |
|  |  | 3rd quartile | 15.3615 | 7.85 | 11.344 | 18.6016 | 13.16 |
|  |  | Maximum | 88.4682 | 67.433 | 86.089 | 83.2353 | 88.468 |
| **After QC filtering** | Total number of cells | | 509 | 588 | 595 | 497 | 2,189 |
|  | Average number of genes per cell | | 3,720 | 3,670 | 3,663 | 3,599 | 3,664 |
|  |  | Minimum | 300 | 297 | 303 | 415 | 297 |
|  |  | 1st quartile | 2,880 | 2,925 | 2,552 | 2,979 | 2,829 |
|  |  | Median | 3,829 | 3,878 | 3,826 | 3,680 | 3,828 |
|  |  | 3rd quartile | 4,609 | 4,596 | 4,780 | 4,395 | 4,581 |
|  |  | Maximum | 6,459 | 6,411 | 6,496 | 6,387 | 6,496 |
|  | Average number of reads per cell | | 16,004 | 18,045 | 16,844 | 15,856 | 16,747 |
|  |  | Minimum | 572 | 516 | 507 | 591 | 507 |
|  |  | 1st quartile | 9,342 | 10,557 | 9,180 | 9,838 | 9,608 |
|  |  | Median | 14,946 | 17,315 | 15,315 | 14,497 | 15,414 |
|  |  | 3rd quartile | 21,191 | 23,692 | 22,720 | 20,678 | 22,282 |
|  |  | Maximum | 46,574 | 53,604 | 47,169 | 42,418 | 53,604 |
|  | Average percent of mitochondria genes per cell | | 8.6894 | 5.687 | 7.372 | 8.6976 | 7.527 |
|  |  | Minimum | 0.1506 | 0 | 0 | 0.0738 | 0 |
|  |  | 1st quartile | 6.557 | 3.833 | 5.564 | 5.7253 | 5.258 |
|  |  | Median | 8.0043 | 5.419 | 7.066 | 7.83 | 7.01 |
|  |  | 3rd quartile | 10.5522 | 7.045 | 8.867 | 11.6118 | 9.303 |
|  |  | Maximum | 19.8544 | 18.856 | 19.236 | 19.8853 | 19.885 |

**Table S3**. Summary table of the number and the percent of cells in each cluster and in each treatment.

| **Cluster ID** | **PBS** | | | **LPS** | | | **4PBA** | | | **LPS4PBA** | | | **Total per cluster** | |
| --- | --- | --- | --- | --- | --- | --- | --- | --- | --- | --- | --- | --- | --- | --- |
|  | # cells | % in cluster | % in treatment | # cells | % in cluster | % in treatment | # cells | % in cluster | % in treatment | # cells | % in cluster | % in treatment | # cells per cluster | % from total |
| **0** | 359 | 95% | 72% | 17 | 5% | 3% | 1 | 0% | 0% | - | 0% | 0% | 377 | 17% |
| **1** | 13 | 4% | 3% | 69 | 21% | 12% | 132 | 41% | 26% | 111 | 34% | 19% | 325 | 15% |
| **2** | 1 | 0% | 0% | 16 | 7% | 3% | 74 | 31% | 15% | 147 | 62% | 25% | 238 | 11% |
| **3** | 3 | 1% | 1% | 4 | 2% | 1% | 104 | 45% | 20% | 121 | 52% | 20% | 232 | 11% |
| **4** | 19 | 9% | 4% | 190 | 90% | 32% | - | 0% | 0% | 2 | 1% | 0% | 211 | 10% |
| **5** | 23 | 12% | 5% | 165 | 85% | 28% | 1 | 1% | 0% | 5 | 3% | 1% | 194 | 9% |
| **6** | 37 | 20% | 7% | 82 | 45% | 14% | 9 | 5% | 2% | 53 | 29% | 9% | 181 | 8% |
| **7** | 10 | 6% | 2% | 2 | 1% | 0% | 104 | 62% | 20% | 52 | 31% | 9% | 168 | 8% |
| **8** | 3 | 2% | 1% | 6 | 4% | 1% | 62 | 41% | 12% | 81 | 53% | 14% | 152 | 7% |
| **9** | 27 | 39% | 5% | 20 | 29% | 3% | 14 | 20% | 3% | 9 | 13% | 2% | 70 | 3% |
| **10** | 2 | 5% | 0% | 17 | 41% | 3% | 8 | 20% | 2% | 14 | 34% | 2% | 41 | 2% |
| **Total # cells per treatment & % from total** | 497 | 23% |  | 588 | 27% |  | 509 | 23% |  | 595 | 27% |  | 2,189 |  |

**Table S4.** Statistics of positive and negative gene sets per cluster.

| **Gene Set^*^** | **The number of genes in a gene set type** | **Cluster 0** | **Cluster 1** | **Cluster 2** | **Cluster 3** | **Cluster 4** | **Cluster 5** | **Cluster 6** | **Cluster 7** | **Cluster 8** | **Cluster 9** | **Cluster 10** |
| --- | --- | --- | --- | --- | --- | --- | --- | --- | --- | --- | --- | --- |
| **(a+b)** | The number of DEG | 290 | 271 | 371 | 75 | 240 | 97 | 275 | 229 | 131 | 217 | 178 |
| **(b) Positive gene set** | The number of DEG with OCRs | 165 | 184 | 249 | 43 | 132 | 59 | 161 | 134 | 62 | 150 | 96 |
| **(c+d)** | The number of non-DEG | 4,791 | 4,680 | 5,049 | 7,053 | 6,084 | 6,849 | 893 | 6,177 | 4,497 | 7,965 | 7,220 |
| **(c)** | The number of non-DEG without OCRs | 1,614 | 1,649 | 1,746 | 2,458 | 2,089 | 2,305 | 298 | 2,145 | 1,507 | 2,845 | 2,485 |
| **Subset of (c), Negative gene set 1** | The number of non-DEG without OCRs with motifs | 1,479 | 1,508 | 1,597 | 2,261 | 1,916 | 2,123 | 273 | 1,988 | 1,386 | 2,593 | 2,276 |
| **(d) Negative gene set 2** | The number of non-DEG with OCRs | 3,177 | 3,031 | 3,303 | 4,595 | 3,995 | 4,544 | 595 | 4,032 | 2,990 | 5,120 | 4,735 |

* Gene sets (a, b, c, d) were defined in Step 1 in **Figure S3**.

**Table S5.** List of top 10 motifs.

| **Method** | **Random Forest** | | **Chi-squared Test** | |
| --- | --- | --- | --- | --- |
| **Rank** | **Top 10 motifs** | **Importance Score** | **Top 10 motifs** | **-Log_10_P-value** |
| **Cluster 0** | | | | |
| 1 | ETV5_MOUSE.H11MO.0.D | 43.44 | IRF4_MOUSE.H11MO.0.A | 0.00E+00 |
| 2 | SP4_MOUSE.H11MO.1.B | 18.45 | SPIB_MOUSE.H11MO.0.A | 0.00E+00 |
| 3 | IRF1_MOUSE.H11MO.0.A | 11.83 | SPI1_MOUSE.H11MO.0.A | 5.79E-298 |
| 4 | ARI3A_MOUSE.H11MO.0.D | 10.58 | IRF8_MOUSE.H11MO.0.A | 1.07E-270 |
| 5 | IRF3_MOUSE.H11MO.0.A | 8.74 | SP1_MOUSE.H11MO.0.A | 3.35E-143 |
| 6 | SP4_MOUSE.H11MO.0.B | 6.56 | FUBP1_MOUSE.H11MO.0.D | 4.09E-113 |
| 7 | KLF3_MOUSE.H11MO.0.A | 6.34 | IRF1_MOUSE.H11MO.0.A | 2.34E-112 |
| 8 | IRF4_MOUSE.H11MO.0.A | 6.13 | SP2_MOUSE.H11MO.0.B | 4.23E-101 |
| 9 | KLF15_MOUSE.H11MO.0.A | 5.87 | ARI3A_MOUSE.H11MO.0.D | 2.31E-96 |
| 10 | STAT2_MOUSE.H11MO.0.A | 5.54 | WT1_MOUSE.H11MO.0.B | 6.44E-84 |
| **Cluster 1** | | | | |
| 1 | SP4_MOUSE.H11MO.0.B | 35.64 | SPIB_MOUSE.H11MO.0.A | 9.75E-131 |
| 2 | ARI3A_MOUSE.H11MO.0.D | 35.33 | SPI1_MOUSE.H11MO.0.A | 8.01E-104 |
| 3 | TCF7_MOUSE.H11MO.0.A | 28.38 | SP2_MOUSE.H11MO.0.B | 3.22E-90 |
| 4 | KLF15_MOUSE.H11MO.0.A | 27.74 | SP1_MOUSE.H11MO.0.A | 2.86E-88 |
| 5 | IRF1_MOUSE.H11MO.0.A | 13.72 | FUBP1_MOUSE.H11MO.0.D | 2.75E-82 |
| 6 | SP4_MOUSE.H11MO.1.B | 10.21 | IRF1_MOUSE.H11MO.0.A | 1.99E-77 |
| 7 | STAT2_MOUSE.H11MO.0.A | 8.98 | IRF4_MOUSE.H11MO.0.A | 4.06E-69 |
| 8 | STAT1_MOUSE.H11MO.0.A | 8.29 | ARI3A_MOUSE.H11MO.0.D | 3.04E-64 |
| 9 | FUBP1_MOUSE.H11MO.0.D | 7.43 | WT1_MOUSE.H11MO.0.B | 2.32E-44 |
| 10 | ZN281_MOUSE.H11MO.0.A | 6.73 | IRF8_MOUSE.H11MO.0.A | 2.14E-41 |
| **Cluster 2** | | | | |
| 1 | TCF7_MOUSE.H11MO.0.A | 44.87 | SPIB_MOUSE.H11MO.0.A | 1.04E-123 |
| 2 | KLF15_MOUSE.H11MO.0.A | 44.14 | SP2_MOUSE.H11MO.0.B | 7.64E-121 |
| 3 | ARI3A_MOUSE.H11MO.0.D | 29.6 | SP1_MOUSE.H11MO.0.A | 3.56E-115 |
| 4 | STAT2_MOUSE.H11MO.0.A | 20.39 | IRF4_MOUSE.H11MO.0.A | 1.10E-113 |
| 5 | SP4_MOUSE.H11MO.0.B | 13.75 | IRF8_MOUSE.H11MO.0.A | 1.55E-109 |
| 6 | IRF3_MOUSE.H11MO.0.A | 11.98 | FUBP1_MOUSE.H11MO.0.D | 3.89E-105 |
| 7 | IRF1_MOUSE.H11MO.0.A | 10.47 | IRF1_MOUSE.H11MO.0.A | 5.30E-97 |
| 8 | SP4_MOUSE.H11MO.1.B | 6.18 | SPI1_MOUSE.H11MO.0.A | 5.43E-91 |
| 9 | RREB1_MOUSE.H11MO.0.D | 5.03 | ARI3A_MOUSE.H11MO.0.D | 1.48E-77 |
| 10 | STAT1_MOUSE.H11MO.0.A | 4.72 | WT1_MOUSE.H11MO.0.B | 2.55E-48 |
| **Cluster 3** | | | | |
| 1 | SPIB_MOUSE.H11MO.0.A | 15.55 | SPIB_MOUSE.H11MO.0.A | 0.00E+00 |
| 2 | STAT2_MOUSE.H11MO.0.A | 10.74 | IRF4_MOUSE.H11MO.0.A | 0.00E+00 |
| 3 | FLI1_MOUSE.H11MO.1.A | 7.96 | SPI1_MOUSE.H11MO.0.A | 0.00E+00 |
| 4 | SP5_MOUSE.H11MO.0.C | 5.39 | IRF8_MOUSE.H11MO.0.A | 7.51E-200 |
| 5 | MAZ_MOUSE.H11MO.0.A | 4.38 | FUBP1_MOUSE.H11MO.0.D | 5.15E-18 |
| 6 | SP4_MOUSE.H11MO.1.B | 3.13 | ARI3A_MOUSE.H11MO.0.D | 5.82E-15 |
| 7 | FUBP1_MOUSE.H11MO.0.D | 1.28 | IRF1_MOUSE.H11MO.0.A | 7.02E-15 |
| 8 | FOXJ3_MOUSE.H11MO.1.B | 0.97 | SP5_MOUSE.H11MO.0.C | 1.73E-10 |
| 9 | IRF1_MOUSE.H11MO.0.A | 0.88 | MAZ_MOUSE.H11MO.0.A | 2.54E-07 |
| 10 |  |  | CTCF_MOUSE.H11MO.0.A | 1.35E-05 |
| **Cluster 4** | | | | |
| 1 | SPI1_MOUSE.H11MO.0.A | 15.22 | SPIB_MOUSE.H11MO.0.A | 0.00E+00 |
| 2 | ETV5_MOUSE.H11MO.0.D | 13.13 | IRF4_MOUSE.H11MO.0.A | 0.00E+00 |
| 3 | STAT1_MOUSE.H11MO.0.A | 12.11 | SPI1_MOUSE.H11MO.0.A | 0.00E+00 |
| 4 | IRF8_MOUSE.H11MO.0.A | 10.05 | IRF8_MOUSE.H11MO.0.A | 0.00E+00 |
| 5 | IRF4_MOUSE.H11MO.0.A | 7.35 | FUBP1_MOUSE.H11MO.0.D | 2.67E-69 |
| 6 | KLF15_MOUSE.H11MO.0.A | 6.89 | IRF1_MOUSE.H11MO.0.A | 5.18E-56 |
| 7 | SP4_MOUSE.H11MO.0.B | 6.49 | ARI3A_MOUSE.H11MO.0.D | 6.83E-54 |
| 8 | ARI3A_MOUSE.H11MO.0.D | 6.02 | SP1_MOUSE.H11MO.0.A | 4.12E-33 |
| 9 | SPIB_MOUSE.H11MO.0.A | 6.01 | MAZ_MOUSE.H11MO.0.A | 1.05E-30 |
| 10 | STAT2_MOUSE.H11MO.0.A | 5.95 | SP5_MOUSE.H11MO.0.C | 1.09E-24 |
| **Cluster 5** | | | | |
| 1 | SPI1_MOUSE.H11MO.0.A | 6.1 | SPIB_MOUSE.H11MO.0.A | 0.00E+00 |
| 2 | KLF15_MOUSE.H11MO.0.A | 5.77 | SPI1_MOUSE.H11MO.0.A | 0.00E+00 |
| 3 | IRF8_MOUSE.H11MO.0.A | 4.83 | IRF4_MOUSE.H11MO.0.A | 0.00E+00 |
| 4 | SPIB_MOUSE.H11MO.0.A | 4.64 | IRF8_MOUSE.H11MO.0.A | 9.54E-154 |
| 5 | STAT1_MOUSE.H11MO.0.A | 4.57 | FUBP1_MOUSE.H11MO.0.D | 1.64E-27 |
| 6 | ARI3A_MOUSE.H11MO.0.D | 4.08 | ARI3A_MOUSE.H11MO.0.D | 8.50E-27 |
| 7 | STAT2_MOUSE.H11MO.0.A | 3.83 | IRF1_MOUSE.H11MO.0.A | 2.03E-25 |
| 8 | IRF3_MOUSE.H11MO.0.A | 3.72 | SP5_MOUSE.H11MO.0.C | 2.07E-22 |
| 9 | PURA_MOUSE.H11MO.0.D | 2.99 | MAZ_MOUSE.H11MO.0.A | 1.18E-17 |
| 10 | SP4_MOUSE.H11MO.0.B | 2.47 | WT1_MOUSE.H11MO.0.B | 1.93E-13 |
| **Cluster 6** | | | | |
| 1 | TCF7_MOUSE.H11MO.0.A | 29.35 | SPIB_MOUSE.H11MO.0.A | 1.18E-74 |
| 2 | SP4_MOUSE.H11MO.1.B | 25.48 | IRF1_MOUSE.H11MO.0.A | 9.86E-71 |
| 3 | SP4_MOUSE.H11MO.0.B | 21.52 | SPI1_MOUSE.H11MO.0.A | 1.82E-68 |
| 4 | STAT2_MOUSE.H11MO.0.A | 11.56 | FUBP1_MOUSE.H11MO.0.D | 4.68E-68 |
| 5 | IRF1_MOUSE.H11MO.0.A | 10.26 | ARI3A_MOUSE.H11MO.0.D | 2.44E-57 |
| 6 | ARI3A_MOUSE.H11MO.0.D | 8.15 | SP5_MOUSE.H11MO.0.C | 1.90E-47 |
| 7 | ZBT17_MOUSE.H11MO.0.A | 6.15 | WT1_MOUSE.H11MO.0.B | 1.41E-31 |
| 8 | IRF3_MOUSE.H11MO.0.A | 5.71 | SP1_MOUSE.H11MO.0.A | 2.18E-29 |
| 9 | MAZ_MOUSE.H11MO.0.A | 2.88 | STAT2_MOUSE.H11MO.0.A | 1.21E-28 |
| 10 |  |  | STAT1_MOUSE.H11MO.0.A | 8.10E-26 |
| **Cluster 7** | | | | |
| 1 | ETV5_MOUSE.H11MO.0.D | 27.9 | SPIB_MOUSE.H11MO.0.A | 0.00E+00 |
| 2 | STAT1_MOUSE.H11MO.0.A | 21.81 | SPI1_MOUSE.H11MO.0.A | 0.00E+00 |
| 3 | KLF15_MOUSE.H11MO.0.A | 9.51 | IRF4_MOUSE.H11MO.0.A | 0.00E+00 |
| 4 | SP4_MOUSE.H11MO.0.B | 8.17 | IRF8_MOUSE.H11MO.0.A | 0.00E+00 |
| 5 | IRF3_MOUSE.H11MO.0.A | 8.08 | SP1_MOUSE.H11MO.0.A | 1.32E-98 |
| 6 | SPI1_MOUSE.H11MO.0.A | 7.66 | FUBP1_MOUSE.H11MO.0.D | 2.01E-74 |
| 7 | IRF4_MOUSE.H11MO.0.A | 7.02 | IRF1_MOUSE.H11MO.0.A | 9.35E-69 |
| 8 | SP3_MOUSE.H11MO.0.B | 6.01 | ARI3A_MOUSE.H11MO.0.D | 3.21E-66 |
| 9 | ARI3A_MOUSE.H11MO.0.D | 4.86 | SP2_MOUSE.H11MO.0.B | 4.76E-56 |
| 10 | STAT2_MOUSE.H11MO.0.A | 4.42 | WT1_MOUSE.H11MO.0.B | 2.80E-44 |
| **Cluster 8** | | | | |
| 1 | ETV5_MOUSE.H11MO.0.D | 15.98 | SPIB_MOUSE.H11MO.0.A | 2.33E-112 |
| 2 | SP1_MOUSE.H11MO.1.A | 11.13 | SPI1_MOUSE.H11MO.0.A | 1.60E-89 |
| 3 | KLF15_MOUSE.H11MO.0.A | 9.79 | IRF4_MOUSE.H11MO.0.A | 5.64E-46 |
| 4 | IRF3_MOUSE.H11MO.0.A | 6.74 | STAT1_MOUSE.H11MO.0.A | 6.78E-44 |
| 5 | ARI3A_MOUSE.H11MO.0.D | 6.68 | IRF1_MOUSE.H11MO.0.A | 4.50E-38 |
| 6 | STAT2_MOUSE.H11MO.0.A | 5.21 | SP1_MOUSE.H11MO.0.A | 2.07E-37 |
| 7 | MAZ_MOUSE.H11MO.0.A | 4.09 | ARI3A_MOUSE.H11MO.0.D | 1.88E-32 |
| 8 | TCF7_MOUSE.H11MO.0.A | 4.01 | SP2_MOUSE.H11MO.0.B | 3.30E-32 |
| 9 | SP5_MOUSE.H11MO.0.C | 3.42 | WT1_MOUSE.H11MO.0.B | 3.13E-31 |
| 10 | FOXJ3_MOUSE.H11MO.0.A | 3.21 | IRF3_MOUSE.H11MO.0.A | 3.99E-29 |
| **Cluster 9** | | | | |
| 1 | ETV5_MOUSE.H11MO.0.D | 20.3 | IRF4_MOUSE.H11MO.0.A | 4.62E-107 |
| 2 | ARI3A_MOUSE.H11MO.0.D | 19.38 | SP1_MOUSE.H11MO.0.A | 8.18E-96 |
| 3 | TCF7_MOUSE.H11MO.0.A | 16.01 | SP2_MOUSE.H11MO.0.B | 6.02E-92 |
| 4 | SP4_MOUSE.H11MO.0.B | 14.37 | IRF8_MOUSE.H11MO.0.A | 1.87E-84 |
| 5 | KLF15_MOUSE.H11MO.0.A | 10.87 | FUBP1_MOUSE.H11MO.0.D | 9.10E-80 |
| 6 | STAT2_MOUSE.H11MO.0.A | 7.28 | IRF1_MOUSE.H11MO.0.A | 6.55E-77 |
| 7 | FUBP1_MOUSE.H11MO.0.D | 7.06 | ARI3A_MOUSE.H11MO.0.D | 1.70E-67 |
| 8 | SP1_MOUSE.H11MO.0.A | 6.36 | WT1_MOUSE.H11MO.0.B | 7.28E-50 |
| 9 | IRF4_MOUSE.H11MO.0.A | 6.33 | STAT2_MOUSE.H11MO.0.A | 2.71E-37 |
| 10 | IRF1_MOUSE.H11MO.0.A | 5.74 | STAT1_MOUSE.H11MO.0.A | 3.94E-29 |
| **Cluster 10** | | | | |
| 1 | ETV5_MOUSE.H11MO.0.D | 23.39 | SPIB_MOUSE.H11MO.0.A | 0.00E+00 |
| 2 | KLF15_MOUSE.H11MO.0.A | 17.14 | SPI1_MOUSE.H11MO.0.A | 0.00E+00 |
| 3 | STAT2_MOUSE.H11MO.0.A | 12.79 | IRF4_MOUSE.H11MO.0.A | 2.03E-117 |
| 4 | SPI1_MOUSE.H11MO.0.A | 11.22 | IRF8_MOUSE.H11MO.0.A | 2.03E-99 |
| 5 | SP3_MOUSE.H11MO.0.B | 10.3 | SP1_MOUSE.H11MO.0.A | 5.71E-47 |
| 6 | ARI3A_MOUSE.H11MO.0.D | 9.13 | FUBP1_MOUSE.H11MO.0.D | 1.62E-46 |
| 7 | TCF7_MOUSE.H11MO.0.A | 6.36 | IRF1_MOUSE.H11MO.0.A | 1.52E-44 |
| 8 | SP1_MOUSE.H11MO.1.A | 5.85 | ARI3A_MOUSE.H11MO.0.D | 4.71E-35 |
| 9 | ELF5_MOUSE.H11MO.0.A | 5.24 | SP2_MOUSE.H11MO.0.B | 8.77E-30 |
| 10 | SALL1_MOUSE.H11MO.0.D | 5.21 | WT1_MOUSE.H11MO.0.B | 1.24E-23 |

**Table S6.** Motif profile of top 10 motifs from 11 clusters by RF and CT. The motif profile provides motif model names, frequency of the motifs across 11 clusters, TF family names, and motif logos. The original motif model names included “mouse.H11MO” which indicates the source of species and the version of HOCOMOCO database. A list of motif models was ordered by transcription factor family.

| **Method** | **Motif model** | **Freq** | **TF family** | **Motif sequence logo** |
| --- | --- | --- | --- | --- |
| RF | ARI3A.0.D | 10 | ARID-related factors {3.7.1} | 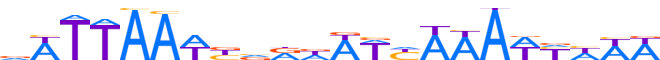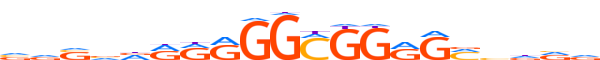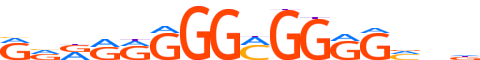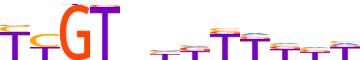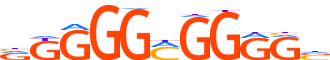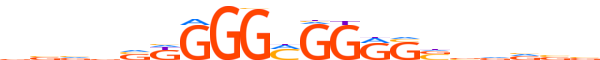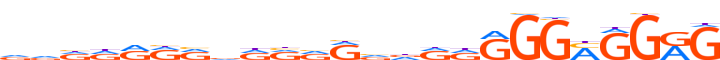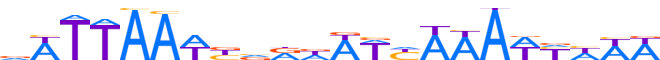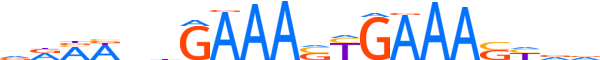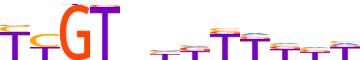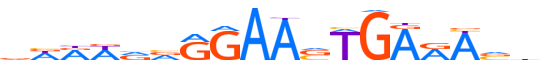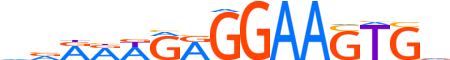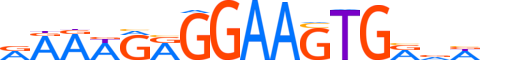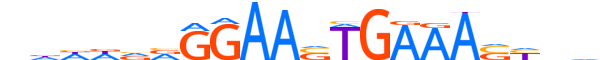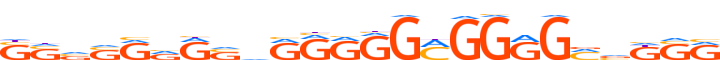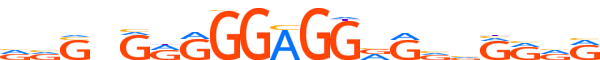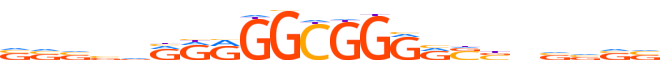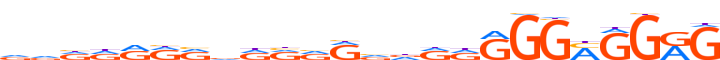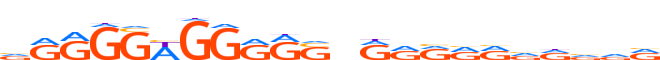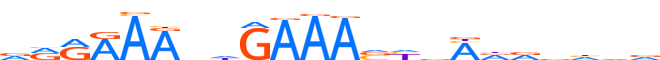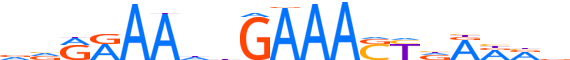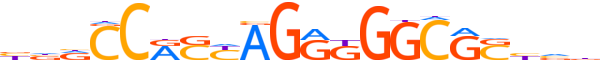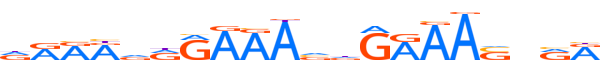 |
| RF | ELF5.0.A | 1 | Ets-related factors {3.5.2} | 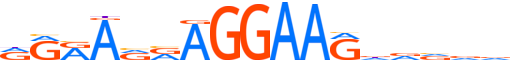 |
| RF | ETV5.0.D | 6 | Ets-related factors {3.5.2} | 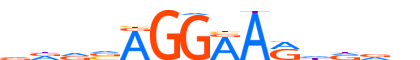 |
| RF | FLI1.1.A | 1 | Ets-related factors {3.5.2} | 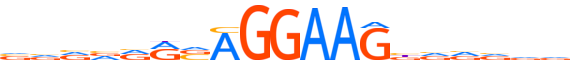 |
| RF | SPI1.0.A | 4 | Ets-related factors {3.5.2} | 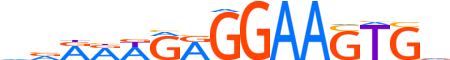 |
| RF | SPIB.0.A | 3 | Ets-related factors {3.5.2} | 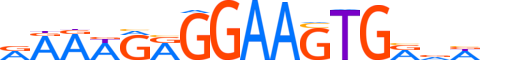 |
| RF | MAZ.0.A | 3 | Factors with multiple dispersed zinc fingers {2.3.4} | 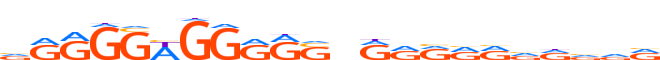 |
| RF | RREB1.0.D | 1 | Factors with multiple dispersed zinc fingers {2.3.4} | 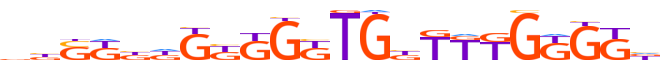 |
| RF | SALL1.0.D | 1 | Factors with multiple dispersed zinc fingers {2.3.4} | 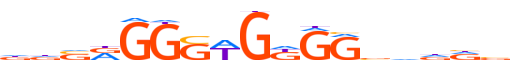 |
| RF | ZBT17.0.A | 1 | Factors with multiple dispersed zinc fingers {2.3.4} | 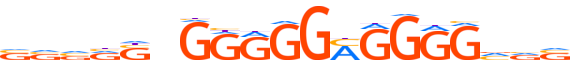 |
| RF | FOXJ3.0.A | 1 | Forkhead box (FOX) factors {3.3.1} | 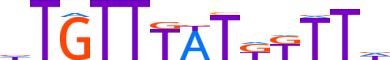 |
| RF | FOXJ3.1.B | 1 | Forkhead box (FOX) factors {3.3.1} | 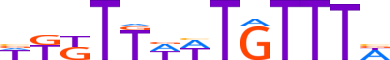 |
| RF | IRF1.0.A | 6 | Interferon-regulatory factors {3.5.3} | 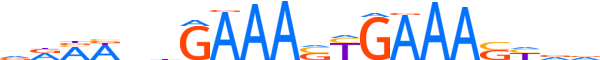 |
| RF | IRF3.0.A | 6 | Interferon-regulatory factors {3.5.3} | 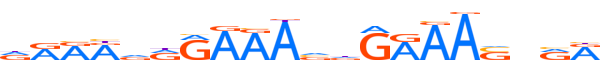 |
| RF | IRF4.0.A | 4 | Interferon-regulatory factors {3.5.3} | 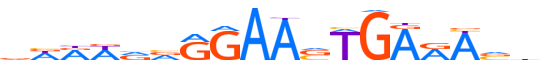 |
| RF | IRF8.0.A | 2 | Interferon-regulatory factors {3.5.3} | 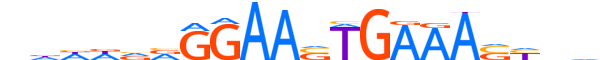 |
| RF | ZN281.0.A | 1 | More than 3 adjacent zinc finger factors {2.3.3} | 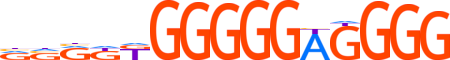 |
| RF | PURA.0.D | 1 | PUR {0.0.5} | 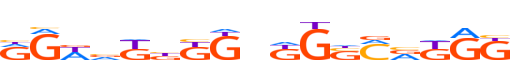 |
| RF | STAT1.0.A | 5 | STAT factors {6.2.1} | 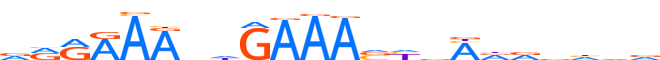 |
| RF | STAT2.0.A | 11 | STAT factors {6.2.1} | 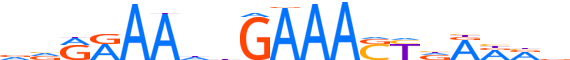 |
| RF | TCF7.0.A | 6 | TCF-7-related factors {4.1.3} | 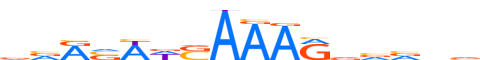 |
| RF | KLF15.0.A | 9 | Three-zinc finger Krüppel-related factors {2.3.1} | 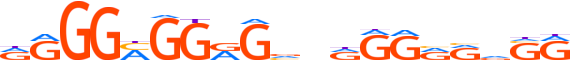 |
| RF | KLF3.0.A | 1 | Three-zinc finger Krüppel-related factors {2.3.1} | 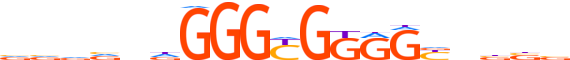 |
| RF | SP1.0.A | 1 | Three-zinc finger Krüppel-related factors {2.3.1} | 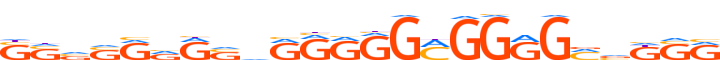 |
| RF | SP1.1.A | 2 | Three-zinc finger Krüppel-related factors {2.3.1} | 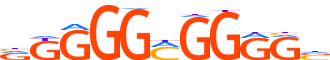 |
| RF | SP3.0.B | 2 | Three-zinc finger Krüppel-related factors {2.3.1} | 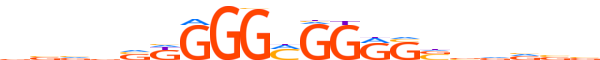 |
| RF | SP4.0.B | 8 | Three-zinc finger Krüppel-related factors {2.3.1} | 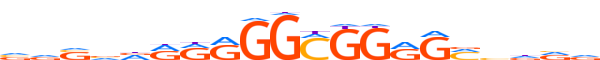 |
| RF | SP4.1.B | 5 | Three-zinc finger Krüppel-related factors {2.3.1} | 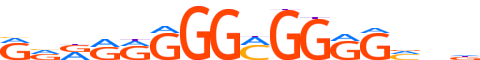 |
| RF | SP5.0.C | 2 | Three-zinc finger Krüppel-related factors {2.3.1} | 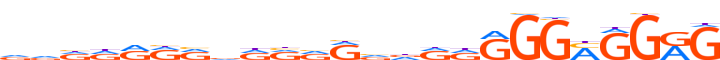 |
| RF | FUBP1.0.D | 3 |  | 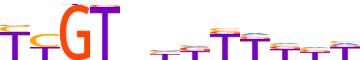 |
| CT | ARI3A.0.D | 11 | ARID-related factors {3.7.1} | 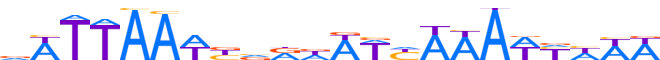 |
| CT | SPI1.0.A | 10 | Ets-related factors {3.5.2} | 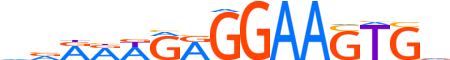 |
| CT | SPIB.0.A | 10 | Ets-related factors {3.5.2} | 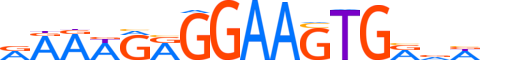 |
| CT | MAZ.0.A | 3 | Factors with multiple dispersed zinc fingers {2.3.4} | 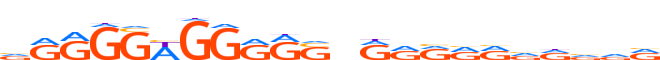 |
| CT | IRF1.0.A | 11 | Interferon-regulatory factors {3.5.3} | 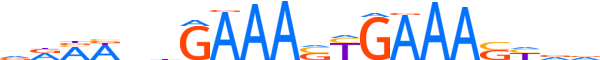 |
| CT | IRF3.0.A | 1 | Interferon-regulatory factors {3.5.3} | 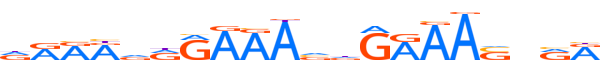 |
| CT | IRF4.0.A | 10 | Interferon-regulatory factors {3.5.3} | 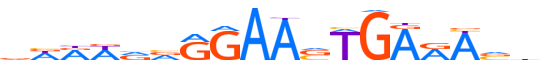 |
| CT | IRF8.0.A | 9 | Interferon-regulatory factors {3.5.3} | 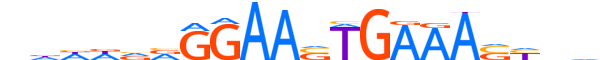 |
| CT | CTCF.0.A | 1 | More than 3 adjacent zinc finger factors {2.3.3} | 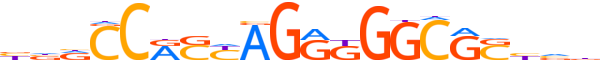 |
| CT | WT1.0.B | 9 | More than 3 adjacent zinc finger factors {2.3.3} | 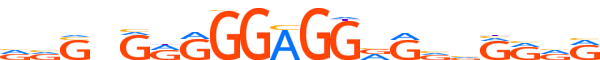 |
| CT | STAT1.0.A | 3 | STAT factors {6.2.1} | 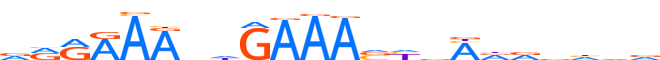 |
| CT | STAT2.0.A | 2 | STAT factors {6.2.1} | 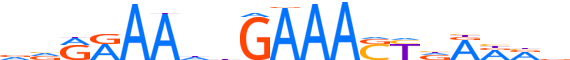 |
| CT | SP1.0.A | 9 | Three-zinc finger Krüppel-related factors {2.3.1} | 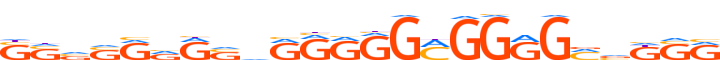 |
| CT | SP2.0.B | 7 | Three-zinc finger Krüppel-related factors {2.3.1} | 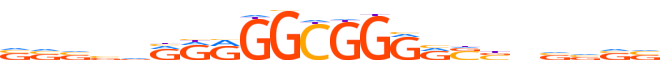 |
| CT | SP5.0.C | 4 | Three-zinc finger Krüppel-related factors {2.3.1} | 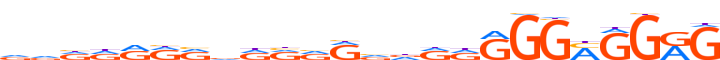 |
| CT | FUBP1.0.D | 10 |  | 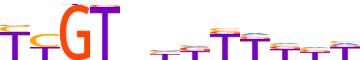 |

**Figure S1.** Quality control metrics. Quality control was preformed and cells with low quality were filtered out. Quality control metrics were generated before filtering cells (A, C) and after filtering cells (B, D). Violin plots (A, B) visualize distribution of the number of genes (# Feature_RNA), the number of UMIs (# Count_RNA), and the percentage of mitochondria genes (percent.mt) detected per cell from four samples. Scatter plots (C, D) visualize relationships between the number of UMIs (# Count_RNA) and the percentage of mitochondria genes (percent.mt) (left) or the number of genes (# Feature_RNA) (right). Correlation values were located on the top of individual scatter plots. Colors of dots indicate identity of cells among four samples.

**Figure S2.** Gene expression of 29 marker genes across samples and clusters.

**Figure S3.** Schematic pipeline of motif enrichment analysis and features selection by Chi-squared test and random forest. Each step was performed for individual clusters respectively. There are four steps. First, we defined input gene sets. We collected a positive gene set (b) among genes that were DEG and on OCR. We set up two negative gene sets. Genes in negative gene set 1 (c) were from non-DEG and on non-OCR. Genes in negative gene set 2 (d) were from non-DEG and on OCR. In detail, differentially expressed genes (DEGs) (a, b) in a cluster were obtained from single cell RNA-seq (scRNA-seq) and the DEGs were genes that were expressed at least 10% of cells in the cluster and statistically significant, and whose fold changes were equal to or bigger than 1.5 (percentage of cells >= 0.1 and adjusted p-value < 0.05 and fold change (the cluster vs. the rest of other groups) >= 1.5). Non-DEGs (c, d) in the cluster were genes that were expressed at least 10% of cells in the cluster and statistically not significant, and whose fold changes were smaller than 1.5 (percentage of cells >= 0.1 and adjusted p-value > 0.1 and absolute fold change (the cluster vs. the rest of other groups) < 1.5). Genes in the positive gene set (b) were statistically significant with high fold change values and that had target genes annotated from ATAC-seq peaks. Genes in the negative gene set 1 (c) were genes that were not statistically significant with lower fold change values and that did not have target genes from ATAC-seq peaks. Genes in the negative gene set 2 (d) were genes that were not statistically significant with lower fold change values and that did have target genes from ATAC-seq peaks. Second, we generated hit count matrixes of the positive gene set and the negative gene sets respectively as input data. We scanned 531 HOCOMOCO11 mouse motifs on promoter regions of genes in the gene sets by using FIMO. A cell of a hit count matrix contained the number of hits of a motif on a promoter region of a gene from the motif search. Third, we used Chi-squared test to calculated p-values from contingency tables of individual motifs. Statistically significant motifs (p-value <0.05) were retained and top 10 motifs sorted by -log_10_(p-value) were used to prioritize motifs and further analysis. Fourth, we ran random forest to obtain importance scores and perform feature selection of motifs. We used 5-fold cross-validation with stratified random sampling to split input data into test-train sets and build random forest models. For each iteration, we conducted sub-4-fold cross-validation on the train set. On training set, parameters of a guided regularized random forest were optimized and a model was built. On validation set, the model was evaluated with 18 different evaluation metrics. Also, selected features by the model were reported together with their importance scores. Among four models from the sub-4-fold cross-validation, a best model was selected based on multiplication of ACC, MCC, and F1. The best model and its selected motifs with importance scores were returned to make prediction on test set by the model. Performance of the best model of an iteration was evaluated by its AUC value. We repeated the described process for all other 4 iterations. The best model of a cluster was chosen based on the maximum AUC value from five iterations. The selected motifs by the best model on validation set were retained and top 10 motifs by RF were sorted by their importance score.

**Figure S4**. Performance comparison of gene sets with negative gene set 1 and negative gene set 2. All 84 input motifs were used for evaluation. Input data was separated into five subsets. For each cluster, gene set 1 indicates input gene sets with negative 1 gene set, and gene set 2 indicates input gene sets with negative 2 gene set, as well as positive gene set. (A) Prediction performance in auROC values from 5-fold cross-validation by RF. (B) Log base 10 of P-values by CT.

**Figure S5.** Enriched motifs by random forest (RF) and chi-squared test (CT). Eleven clusters (Y-axis) were ordered corresponding to the potential cellular transition based on the predicted pseudotime. (A) Motif selection results for 11 clusters by RF (top) and CT (bottom). Total 84 motifs from HOCOMOCOv11 database are listed in the x-axis of the plots. CT detected 59 unique motifs and RF detected 82 unique motifs. If motifs were detected by importance scores by RF or significant p-values by CT at least once across 11 clusters, then the motifs were colored in grey. Top 10 motifs identified by each method were highlighted by purple for RF or green for CT. (B) Expression profile of transcription factor genes corresponding to selected motifs in 11 clusters. Expression levels are scaled and sizes of dots are proportional to the percentage of expressed cells in each cluster. Genes were not included if gene accessions from HOCOMOCOv11 are missing in Ensembl annotation file, or genes were not detected in this single-cell RNA-seq data. Some motifs have different names for genes: KAISO - Zbtb33; TF2L1 - Tfcp2l1; THA11 – Thap11; TYY` - Yy1; ZBT17 – Zbtb17.

**Figure S6.** Prediction performance of 11 clusters in (A) auROC and (B) auPRC with top N motifs from random forest (RF) and Chi-squared test (CT) methods (N = {1, …, 10}). Best models were applied to testing data set with certain numbers of motifs as features. By adding top motifs one by one, prediction performances with the different numbers of motifs were evaluated. Each line represents a prediction performance using a ROC curve (A) or PRC (B) with top N motifs as features..

 **Figure S7.** Advantage of feature selection by RF over CT with top N motifs (N = {1, …, 10}). (A) Improvement of prediction performance of 11 clusters with top N motifs in auROC from random forest (RF) and Chi-squared test (CT) methods. There are five auROC values for each box plot from evaluation results to test sets. A black line inside a boxplot, a red dot, and a black dot(s) above or below whiskers represent a median, a mean, an outlier(s) respectively. Prediction results with top 10 motifs by RF show distinct improvement of auROC values with fewer top motifs. (B) Increase of the number of target genes in positive gene sets with top N motifs. For each cluster, individual bars indicate the number of target genes that can be detected by N^th^ motif from RF or CT methods. A red line indicates the accumulated number of target genes that can be detected by 1~N motifs.
